# Identification of CIITA Regulated Genetic Module Dedicated for Antigen Presentation

**DOI:** 10.1371/journal.pgen.1000058

**Published:** 2008-04-25

**Authors:** Michal Krawczyk, Queralt Seguín-Estévez, Elisa Leimgruber, Peter Sperisen, Christoph Schmid, Philipp Bucher, Walter Reith

**Affiliations:** 1 University of Geneva Medical School, CMU, Geneva, Switzerland; 2 Swiss Institute of Bioinformatics, Swiss Institute for Experimental Cancer Research, Epalinges, Switzerland; The Jackson Laboratory, United States of America

## Abstract

The class II trans-activator CIITA is a transcriptional co-activator required for the expression of Major Histocompatibility Complex (MHC) genes. Although the latter function is well established, the global target-gene specificity of CIITA had not been defined. We therefore generated a comprehensive list of its target genes by performing genome-wide scans employing four different approaches designed to identify promoters that are occupied by CIITA in two key antigen presenting cells, B cells and dendritic cells. Surprisingly, in addition to MHC genes, only nine new targets were identified and validated by extensive functional and expression analysis. Seven of these genes are known or likely to function in processes contributing to MHC-mediated antigen presentation. The remaining two are of unknown function. CIITA is thus uniquely dedicated for genes implicated in antigen presentation. The finding that CIITA regulates such a highly focused gene expression module sets it apart from all other transcription factors, for which large-scale binding-site mapping has indicated that they exert pleiotropic functions and regulate large numbers of genes.

## Author Summary

Most mammalian transcription factors and transcriptional co-activators are believed to regulate the activities of numerous genes fulfilling multiple functions. This pleiotropic role has recently been confirmed directly for several individual factors by large-scale mapping studies aimed at generating comprehensive catalogues of their binding sites in the genome. Until now, all transcription factors, for which such studies have been performed, were found to regulate hundreds or even thousands of genes. We demonstrate, here, that the transcriptional co-activator CIITA (class II transactivator) is an exception to this rule. CIITA is a key regulator of the immune system because it controls the transcription of genes coding for Major Histocompatibility Complex (MHC) class II molecules, which are cell-surface molecules that present peptide antigens to T lymphocytes. To address the possibility that CIITA might exert more widespread functions, we have performed extensive genome-wide searches to establish a comprehensive list of CIITA-regulated genes. Surprisingly, we found that CIITA regulates only a small number of genes, most of which code for proteins implicated directly or indirectly in MHC-mediated antigen presentation. CIITA is thus remarkably dedicated for the regulation of a unique set of functionally related genes constituting a genetic module devoted to a single biological process.

## Introduction

Most mammalian transcription factors are believed to activate numerous genes having diverse functions. Direct support for this pleiotropic nature of transcription factor function has been provided by techniques permitting the unbiased mapping of transcription factor binding sites in substantial segments of genomic DNA, whole chromosomes or entire genomes. These large scale location techniques rely on sequencing or microarray (chip) analysis of genomic DNA sequences that are enriched by chromatin immunoprecipitation (ChIP) experiments. To date, all transcription factors for which these methods have been applied were found to bind to numerous target genes, typically in the order of several hundred to several thousand per genome [1]–[5]. The past few years has witnessed a controversy about whether this general rule of pleiotropic function is also valid for the Major Histocompatibility Complex class II (MHC-II) gene transactivator CIITA (NM_000246).

CIITA was first established to be a key regulator of *MHC-II* genes because it was found to be mutated in an *in vitro* generated B cell line lacking MHC-II expression [6]. Mutations in the *CIITA* gene were next shown to be one of the causes of the Bare Lymphocyte Syndrome (BLS) (MIM number: 600005), a hereditary immunodeficiency disease characterized by the virtually complete absence of MHC-II expression and a significant reduction in MHC class I (MHC-I) expression [6],[7]. In accordance with these genetic findings, CIITA was subsequently shown to be a non-DNA-binding transcriptional co-activator that is essential for expression of the genes encoding the α and β chains of all classical and non-classical MHC-II molecules (HLA-DR, HLA-DP, HLA-DQ, HLA-DM, HLA-DO in humans) as well the gene encoding the Invariant chain (Ii), an accessory molecule controlling intracellular transport and peptide loading of MHC-II molecules [7]–[9]. CIITA was also found to contribute, albeit to a lesser extent, to the transcription of *MHC-I* genes [7]–[12].

All clinical and immunological abnormalities documented in BLS patients can be explained by defects in MHC expression [7]. The same is true for knockout mice carrying mutations in the *CIITA* gene [7],[9]. The absence of other overt phenotypes suggested that CIITA is highly dedicated for the transcription of MHC genes. It therefore came as a major surprise when a growing number of reports suggested that CIITA can affect the expression of numerous genes involved in diverse functions within and outside the immune system. Microarray experiments identified the gene encoding Plexin-A1, which was reported to be activated by CIITA in mouse DC [13], and over 40 genes that were suggested to be up-regulated by CIITA in human B cells and interferon-γ (IFNγ) induced cells [14]. The genes encoding IL-4 and Fas ligand were proposed to be repressed by CIITA in mouse T cells [15]–[18]. Those encoding cathepsin E and IL-10 were suggested to be repressed by CIITA in mouse B cells and/or DC [19],[20] . The genes encoding collagen type I α2, tymidine kinase and cyclin D1 were proposed to be repressed by CIITA in IFNγ induced cells [21],[22]. Finally, microarray experiments identified 16 genes of diverse functions that were proposed to be down-regulated by CIITA in human B cells [14]. Taken together, these reports suggested that CIITA has widespread functions extending beyond its well established role in the control of MHC expression.

The notion that CIITA exerts pleiotropic functions was at odds with the highly specific defects observed in BLS patients and CIITA-deficient mice. To address this discrepancy we set out to define the complete set of CIITA target genes by performing genome-scale ChIP-chip experiments. Our results demonstrate that CIITA is remarkably dedicated for the regulation of genes implicated in MHC-II and MHC-I mediated antigen presentation. Outside of the well established *MHC-II*, *MHC-I* and *Ii* genes, only nine new target genes were identified, seven of which are known or likely to function in processes related to antigen presentation. The finding that CIITA regulates such a highly specialized genetic module sets it apart from all other transcription factors for which large scale mapping of binding sites has been performed.

## Results

### Strategies for Identifying CIITA Target Genes

**Figure 1:**
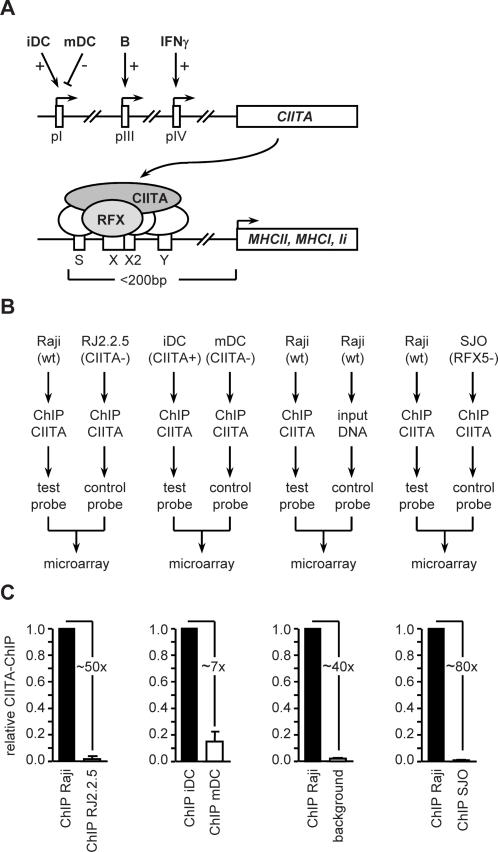
ChIP-chip strategies used to identify target genes of CIITA. (A) Schematic representation of the expression and function of CIITA. Expression of the *CIITA* gene is regulated by three independent promoters – pI, pIII and pIV – that are activated in iDC, B cells and IFNγ induced cells, respectively. pI is silenced after maturation of DC (mDC). CIITA is a non-DNA-binding co-activator that activates expression of its known target genes (*MHC-II*, *MHC-I*, Ii) by binding to a transcription factor complex that assembles on an enhancer consisting of S, X, X2 and Y sequences. Known CIITA target genes contain an S-Y enhancer typically situated less than 200 bp upstream of the transcription initiation site. The X-box-binding factor RFX is essential for CIITA recruitment. (B) Four ChIP-chip strategies were developed for identifying target genes of CIITA in B cells and iDC. Test probes generated from CIITA-ChIP samples obtained from Raji B cells and iDC were hybridized to NimbleGen promoter arrays. Control probes were prepared from input DNA or CIITA-ChIP samples derived from RJ2.2.5 cells, SJO cells or mDC. (C) Enrichment of known CIITA target sequences in CIITA-ChIP probes. Quantitative PCR was used to verify enrichment of the *HLA-DRA* promoter in the indicated test samples (black bars) relative to control samples (open bars). The approximate fold-enrichment is indicated. To estimate enrichment relative to input DNA we used a control sequence exhibiting only nonspecific CIITA association (background). The results show the mean and SD of 3 independent experiments (each performed with triplicate PCR measurements).

Approaches for identifying novel target genes of CIITA were developed on the basis of its expression and mode of action (Figure 1). The pattern of CIITA expression dictates the cell type specificity of MHC-II expression (Figure 1A) [23]. B cells and immature dendritic cells (iDC) are MHC-II positive because they express CIITA. The *CIITA* gene is instead silenced in mature DC (mDC) [24]. Most non-hematopoietic cells are MHC-II negative because they lack CIITA. The latter can however be induced to activate *CIITA* expression by stimulation with IFNγ. CIITA is recruited to its known target genes through protein-protein interactions with a transcription factor complex that assembles on a characteristic enhancer composed of four sequences called the S, X, X2 and Y boxes (Figure 1A) [7]–[9]. Regulatory factor X (RFX) - a trimeric factor containing three subunits called RFX5 (NM_000449), RFXAP (NM_000538) and RFXANK (NM_134440) - is an essential component of this transcription factor complex [25]–[29]. CIITA recruitment is abolished in RFX-deficient cells [30]–[33]. Like defects in CIITA, mutations in the *RFX5*, *RFXAP* and *RFXANK* genes give rise to the BLS disease (MIM number: 601863, 601861, 603200).

Based on the above, we devised genome-wide ChIP-chip approaches according to four different experimental designs (Figure 1B). In all four approaches, ChIP samples obtained with CIITA-specific antibodies were used to prepare probes that were hybridized in conjunction with control probes to microarrays carrying the promoter regions of 27434 human genes. The four strategies differed with respect to the cell type used to prepare the ChIP samples and the control probes to which they were compared. In the first strategy, CIITA-ChIP probes derived from the wild type B cell line Raji were compared with CIITA-ChIP probes derived from RJ2.2.5, a CIITA-deficient mutant of Raji. In the second strategy, CIITA-ChIP samples prepared from iDC were compared with CIITA-ChIP samples from mDC. In the third strategy, CIITA-ChIP probes derived from Raji were compared with input genomic DNA from Raji. In the fourth stratetgy, CIITA-ChIP probes derived from Raji were compared with CIITA-ChIP probes prepared from a cell line (SJO) derived from an RFX5-deficient BLS patient. In all four experimental settings, there is a robust enrichment of known CIITA target sequences, such as the *HLA-DRA* promoter, in the test samples relative to the control samples (Figure 1C).

### Validation of the ChIP-Chip Screens

**Figure 2:**
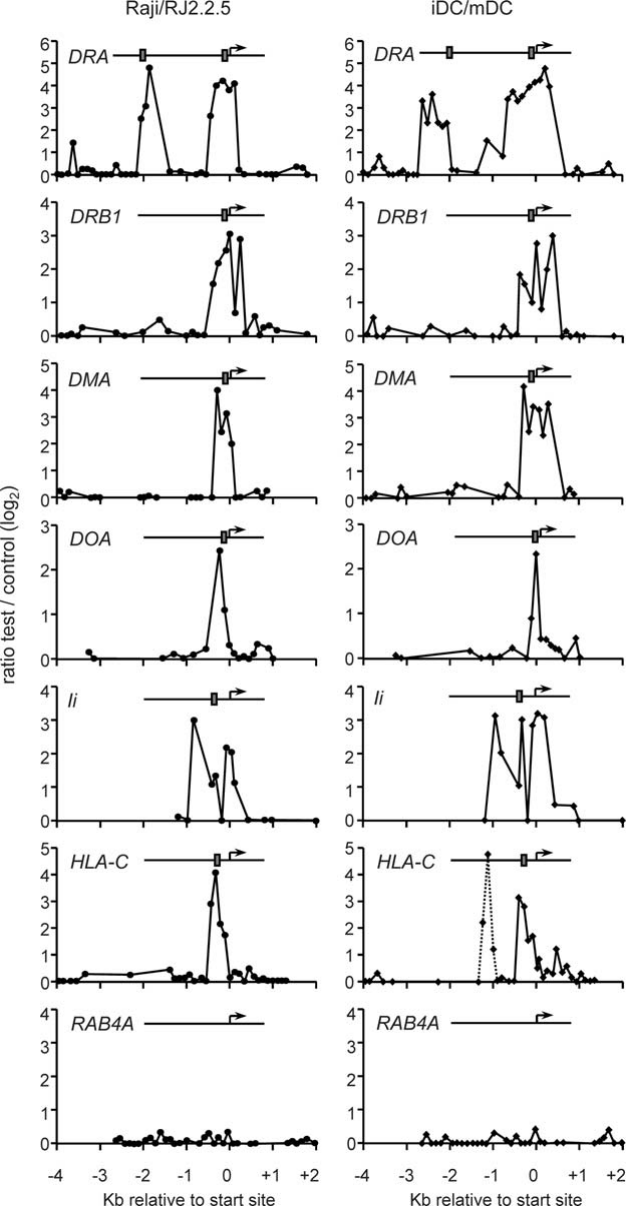
Binding of CIITA to known target genes. Binding of CIITA to the *HLA-DRA*, *HLA-DRB1*, *HLA-DMA*, *HLA-DOA*, *Ii* and *HLA-C* genes is evident in the ChIP-chip experiments performed with Raji B cells and iDC. The *RAB4A* gene was used as negative control. Results are represented as log_2_ ratios between hybridization signals obtained with CIITA-ChIP probes from Raji and RJ2.2.5 (left column) or iDC and mDC (right column). Each dot corresponds to a single oligonucleotide on the array. The dotted line in the right *HLA-C* profile indicates a peak that was not reproduced in all experiments. The schematic maps show positions of the transcription start sites (arrows) and S-Y enhancers (grey boxes). The scale in Kb relative to the start site is provided below.

To validate our screening strategies we examined binding of CIITA to the promoters of well established target genes, including *MHC-II*, *Ii* and *MHC-I* genes (Figure 2, Table 1, Figure S1). Clear binding of CIITA – visualized as peaks in the test/control signal ratios - was observed in Raji B cells and iDC at positions corresponding to the S-Y enhancers of these genes. Despite some variability in the width and height of the peaks, binding of CIITA at the correct position was observed reproducibly in nine independent experiments using all four screening strategies. Binding signals were strong; test/control signal ratios typically ranged from a minimum of 6 to over 30 depending on the target gene and the experiment. Binding of CIITA was never observed at control genes that are not regulated by CIITA (Figure 2, Figure S2). The sensitivity of our ChIP-chip approach was high. The false-negative rate was only ∼5% at well-established target genes (Table 1). Furthermore, robust peaks were observed in all or most experiments even at the *HLA-DOB, HLA-DPB, HLA-DMB* and *HLA-DQA* genes (Table 1) despite the fact that CIITA binding signals observed at these genes in classical ChIP experiments are typically 10–20 fold lower than those observed at the prototypical *HLA-DRA* gene [32].

**Table 1:** Summary of ChIP-chip and classical ChIP experiments.

	gene	accession	ChIP-chip iDC/mDC^1^		ChIP-chip Raji/RJ2.2.5^1^			ChIP-chip Raji/SJO^1^			ChIP-chip Raji/ input	validation by ChIP^2^
			1	2	1	2	3	1	2	3	1	
**MHC-II genes**	*DRA*	NM_019111	**+++**	**+++**	**+++**	**+++**	**+++**	**+++**	**+++**	**+++**	**+++**	**positive**
	*DRB1*	NM_002124	**+++**	**+++**	**+++**	**+++**	**+++**	**+++**	**+++**	**+++**	**+++**	nt
	*DRB5*	NM_002125	**++**	**++**	**++**	**+**	**+**	**++**	**+**	**+**	**++**	nt
	*DQA*	NM_002122	**+**	**++**	**+++**			**+++**			**+++**	**positive**
	*DQB*	NM_002123	**+**	**++**	**++**	**+**	**+**	**++**	**+**	**+**	**++**	nt
	*DOA*	NM_002119	**+++**	**+**	**+++**	**+**	**+**	**+++**	**+**	**+**	**+++**	**positive**
	*DOB*	NM_002120		**++**	**+**	**+++**	**+++**	**+**	**+++**	**+++**	**+**	**positive**
	*DMA*	NM_006120	**+++**	**+++**	**++**	**+++**	**+++**	**+++**	**++**	**++**	**+++**	nt
	*DMB*	NM_002118	**++**	**++**	**+++**	**+**	**+**	**+++**	**+**	**+**	**+++**	**positive**
	*DPA*	NM_033554	**+++**	**+++**	**+++**	**+**	**+**	**+++**	**+**	**+**	**+++**	**positive**
	*DPB*	NM_002121	**+++**	**+++**	**++**	**++**	**++**	**+++**	**++**	**++**	**++**	**positive**
	*Ii*	NM_004355	**+++**	**++**	**++**	**++**	**++**	**++**	**++**	**++**	**+++**	**positive**
**Score 3**	*RAB4B*	NM_016154	**+++**	**+++**	**+**	**+++**	**+++**	**+**	**+++**	**+++**	**++**	**positive**
	*TRIM26*	NM_003449	**++**		**+**	**++**	**++**	**+++**	**+**	**+**	**+++**	**positive**
	*FLJ45422*	NM_001004349	**+**		**+**	**++**	**++**	**+**	**++**	**++**	**+**	**positive**
	*KIAA0841*	BC064390	**+**	**+**		**+**	**+**	**+**		**+**	**+**	**positive**
	*RFX5*	NM_000449	**+**	**+**	**+++**	**++**	**+++**	**++**	**+**	**+**	**++**	**positive**
	*ZNF672*	NM_024836		**++**	**+++**	**+**	**++**	**+**	**++**	**++**	**+**	**positive**
	*MYBPC2*	NM_004533	**+**	**+**		**+**	**+**	**+**		**+**	**+**	**positive**
	*TPP1*	NM_000391	**+**	**++**		**+**	**+**		**+**	**+**	**+**	**positive**
	*SCYL1BP1*	NM_152281	**+**	**++**	**+**	**++**	**+**	**+**	**+**	**+**	**++**	negative
	*BMF*	NM_001003940	**+**		**++**	**++**	**++**	**+++**	**++**	**++**	**++**	negative
	*RALA*	NM_005402		**+**	**+++**	**+**	**+**	**+++**	**+**	**+**	**+++**	negative
	*LRPPRC*	NM_133259		**+**	**+**	**++**	**++**	**++**	**++**	**++**	**++**	negative
**Score2**	*PSMD3*	NM_002809	**+**	**++**	**+**			**+**			**++**	**positive**
	*BRD2*	NM_005104		**+++**		**+**	**+**		**+**	**+**	**+**	negative
	*LOC125893*	BC105738	**+**	**++**		**+**		**+**			**+**	negative
	*TM9SF4*	NM_014742			**+**	**+**	**+**			**+**		negative
	*C1orf151*	NM_001032363	**+++**	**++**	**+**			**+**			**+**	negative
	*MACF1*	NM_033044			**+++**	**+++**	**+++**	**+++**	**+++**	**+++**	**+++**	negative
	*NBPF15*	NM_173638	**+**	**+**		**++**	**+++**			**+++**		nt
	*LEMD2/MLN*	NM_181336			**+++**	**++**	**+**	**++**		**+**	**+++**	negative
	*TRIM14*	NM_033221			**+**	**++**	**+**	**++**	**+**	**+**	**++**	negative
	*AF289566*	AF289566	**+**	**+**		**+**	**+**		**+**	**+**		nt
	*NBPF1*	NM_017940	**+**	**++**		**++**	**+++**		**++**	**++**		nt
	*BC034418*	BC034418	**+**	**+**		**+**	**++**			**+++**	**+**	nt
	*AB007893*	NM_015216	**+++**	**+**	**+**			**++**			**++**	negative
	*DENND1A*	NM_020946	**+**	**++**	**++**			**++**			**+++**	negative
	*CHML*	NM_001821	**++**	**+**	**+++**			**+++**			**+++**	negative
	*ZFYVE19*	NM_001077268	**+**			**+++**	**+++**		**+++**	**+++**		negative
	*ANKRA2*	NM_023039	**++**	**++**	**++**			**++**			**+++**	negative
	*RUFY2*	NM_017987			**+++**	**++**	**+++**	**+**	**+++**	**+++**		negative
	*TDH*	AY101187			**+++**	**++**	**++**	**+++**	**+**	**+**	**+++**	negative
	*FLJ44082*	AK126070			**++**	**+**	**+**	**++**	**+**	**+**	**++**	negative

1 Results of independent CIITA-ChIP-chip experiments performed with NimbleGen promoter arrays: + signs indicate the presence of a peak in the signal ratios; the number of + signs refers to the quality of the peaks; +++ indicates strong signals (log_2_ ratio > 2) at four or more adjacent oligonucleotides or weaker signals at more than four adjacent oligonnucleotides; ++ indicates strong signals at three adjacent oligonucleotides or weaker signals at four adjacent oligonucleotides; + indicates strong signals at two adjacent oligonucleotides or weaker signals at three adjacent oligonucleotides.

2 Results of classical CIITA-ChIP experiments: positive, confirmed binding; negative, absence of binding; nt, not tested.

Certain peaks exhibit a dip in CIITA binding at a position that coincides with the S-Y enhancer (Figure 2). These dips are likely to be artifacts because they generally concerned only a single oligonucleotide on the array, were not observed in all experiments and were not observed in experiments using high density microarrays (see discussion).

### Identification of New CIITA Target Genes

To identify potential new targets of CIITA, we developed a procedure to screen for the presence of reproducible peaks in the test/control signal ratios obtained in multiple Raji/RJ2.2.5 and iDC/mDC comparisons (see Materials and Methods). Candidate genes were assigned a score on the basis of peak height, width and reproducibility. A score of 3 was assigned to the candidate genes at which peak quality and reproducibility were similar to those observed for known target genes (Table 1). A score of 2 was assigned to candidate genes for which peak quality and/or reproducibility were promising but significantly lower than for known target genes (Table 1). Finally a score of 1 was assigned to possible but unlikely candidates exhibiting only weak and poorly reproducible peaks (Table S1). The number of likely candidates was surprisingly low: scores of 3 and 2 were assigned, respectively, to only twelve and twenty genes.

**Figure 3:**
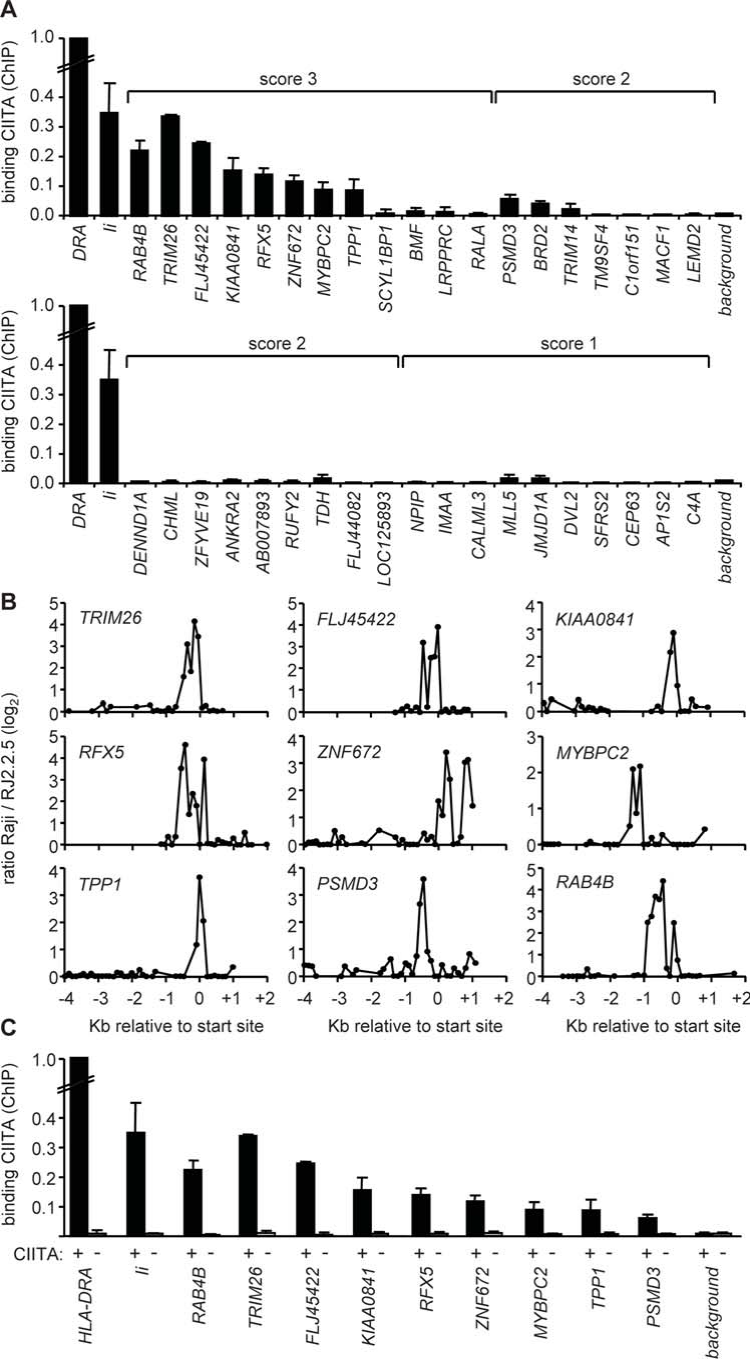
Identification of novel target genes of CIITA. (A) Binding of CIITA to the indicated genes was assessed in Raji B cells by quantitative ChIP. The *HLA-DRA* and *Ii* genes were used as positive controls. As negative control we used a sequence exhibiting only nonspecific CIITA association (background). Scores assigned to the candidate target genes (see Materials and Methods, Tables S1 and S2) are indicated above. Results are expressed relative to binding of CIITA at *HLA-DRA*, and show the mean and SD of 3 independent experiments (each performed with triplicate PCR measurements). Binding was deemed significant if it was greater than 2-fold above background. (B) Representative CIITA-binding profiles obtained in ChIP-chip experiments are provided for the nine new target genes validated in panel A. Results are represented as in Figure 2 for ChIP-chip experiments comparing Raji and RJ2.2.5 cells. (C) Binding of CIITA to the new target genes was confirmed by quantitative ChIP experiments performed with Raji and RJ2.2.5 cells. Positive (*HLA-DRA*, *Ii*) and negative (background) controls were as in panel A. Results are expressed relative to binding of CIITA at *HLA-DRA* in Raji, and show the mean and SD of 3 independent experiments (each performed with triplicate PCR measurements).

To validate bona fide new target genes we screened the most promising candidates for binding of CIITA in Raji B cells by quantitative ChIP experiments (Figure 3A). All twelve score 3 candidates, sixteen score 2 candidates and a selection of the best score 1 candidates were tested. The primers used for real-time PCR analysis of the ChIP experiments were designed within the regions at which peaks were observed in the ChIP-chip experiments. Four score 2 candidates were not tested because suitable primers could not be designed. Binding of CIITA was confirmed for eight score 3 candidates (*RAB4B*, *TRIM26*, *FLJ45422*, *KIAA0841*, *RFX5*, *ZNF672*, *TPP1* and *MYBPC2*) and only one score 2 candidate (*PSMD3*). Weaker binding was observed at two additional score 2 genes (*BRD2* and *TRIM14*), but these were not studied further because signals were only 2–3 fold above background. No significant binding was evident at the remaining score 3 and score 2 genes, or at any of the tested score 1 genes (Figure 3, Table 1, Table S1). The finding that most of the score 3 candidates, only one of the score 2 candidates and none of the tested score 1 candidates are true targets of CIITA demonstrates the validity of our scoring procedure. These results also demonstrate that the specificity of our approach is greatest if peak reproducibility is chosen as the most critical parameter for predicting target genes (Table 1). Thus, among genes at which peaks are present in at least four out of five experiments (MHC-II and score 3 genes) less than 20% were found to be false positives, whereas the false positive rate increased dramatically among genes at which peaks were detected at only three out of five experiments (score 2 genes).

Representative ChIP-chip profiles corresponding to binding of CIITA in Raji are provided in Figure 3B for the nine validated target genes. Peaks similar in quality to those found at the *MHC-II*, *MHC-I* and *Ii* genes are observed near the transcription initiation site of all nine genes (compare Figure 3B with Figure 2 and Figure S1).

To further confirm the specificity of our quantitative ChIP experiments we compared occupation of the new targets by CIITA between Raji and its CIITA-deficient derivative RJ2.2.5. As observed for the control *HLA-DRA* and *Ii* genes, binding of CIITA is reduced to non-specific background levels in RJ2.2.5 at all nine new target genes (Figure 3C).

To address the possibility that numerous true target genes might have been missed by our data analysis procedure we used two additional independent methods to analyze the same datasets. First, the windowing and threshold program ACME (Algorithm for Capturing Microarray Enrichment) [34] was used for peak detection, and significant peaks present in at least four out of five experiments were identified. In addition to MHC-II and related genes, this procedure identified only 17 potential new target genes (data not shown). Quantitative ChIP experiments performed with 11 of these showed that only 3 represented true CIITA targets (*RFX5*, *RAB4B*, *PSMD3*). The other 8 were found to be false positives that had been assigned scores of 3 (3 genes), 2 (2 genes) and 1 (3 genes) by our initial procedure. The remaining 6 candidates were not tested because visual inspection of their ChIP-chip peaks revealed that they were of very low quality and highly likely to correspond to false positive hits. Importantly, 6 of the validated new CIITA target genes (*TRIM26*, *FLJ45422*, *KIAA0841*, *ZNF672*, *TPP1* and *MYBPC2*) were not picked up.

As a second alternative approach we developed an unsupervised clustering procedure (see Material and Methods). This method grouped potential candidates into 8 groups on the basis of peak reproducibility and log_2_ signal ratios (data not shown). The two groups corresponding to the most likely targets contained only 12 and 29 genes respectively. 12 of these were MHC-II genes and 6 were among the new validated targets (*RAB4B*, *TRIM26*, *KIAA0841*, *RFX5*, *ZNF672* and *MYBPC2*). Among the remaining 23 genes, 2 were found to be false positives by ChIP experiments and 22 were eliminated as good candidates by visual inspection of the peaks. Finally, 3 of the newly validated target genes (*FLJ45422*, *TPP1* and *MYBPC2*) were again not singled out as likely candidates. In conclusion, neither alternative approach was superior to our original method with respect to specificity or sensitivity. More importantly, the alternative methods did not identify a large number of likely targets that were missed by our original procedure.

### Binding of CIITA to the New Targets in DC and IFNγ Induced Cells

**Figure 4:**
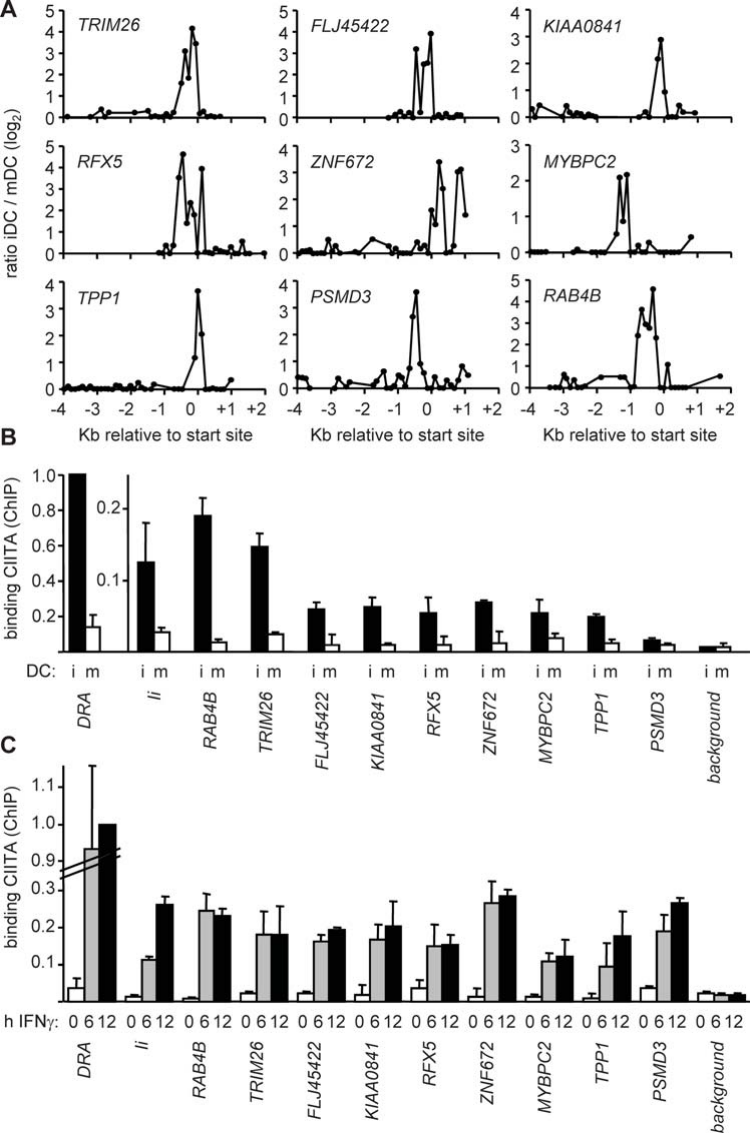
CIITA binds to the new target genes in DC and IFNγ induced cells. (A) Representative CIITA-binding profiles obtained for the new target genes in ChIP-chip experiments performed with DC. Results are represented as in Figure 2 for ChIP-chip experiments comparing iDC and mDC. (B) Binding of CIITA to the new target genes was confirmed by quantitative ChIP experiments performed with iDC and mDC. Results are expressed relative to binding of CIITA at *HLA-DRA* in iDC, and show the mean and SD of 3 independent experiments (each performed with triplicate PCR measurements). (C) Binding of CIITA to the new target genes was confirmed by quantitative ChIP experiments performed with Me67.8 melanoma cells stimulated for 0, 6 or 12 hours with IFNγ. Results are expressed relative to the plateau level obtained at *HLA-DRA* in cells induced for 12 hours, and show the mean and SD of 3 independent experiments (each performed with triplicate PCR measurements). Positive (*HLA-DRA*, *Ii*) and negative (background) controls were as in Figure 3.

Strong and reproducible peaks were also evident at the new target genes in ChIP-chip experiments comparing ChIP probes from iDC and mDC (Figure 4A, Table 1). We therefore performed quantitative ChIP experiments to measure binding of CIITA to the new targets in iDC. For eight of the new targets, significant binding was observed in iDC (Figure 4B). As observed for the control *HLA-DRA* and *Ii* genes, this binding was strongly reduced in mDC (Figure 4B).

CIITA can be induced in most CIITA negative cells by stimulation with IFNγ (Figure 1A). We therefore performed quantitative ChIP experiments to determine whether binding of CIITA to the new targets is induced by IFNγ in a melanoma cell line exhibiting well documented IFNγ induced CIITA expression [35]. IFNγ induced occupation by CIITA was evident at all nine new target genes (Figure 4C). As observed for the control *HLA-DRA* and *Ii* genes, this occupation by CIITA is induced rapidly, reaching maximal levels by 6 hours of stimulation (Figure 4C).

### Recruitment of CIITA to the New Targets Is Dependent on RFX

**Figure 5:**
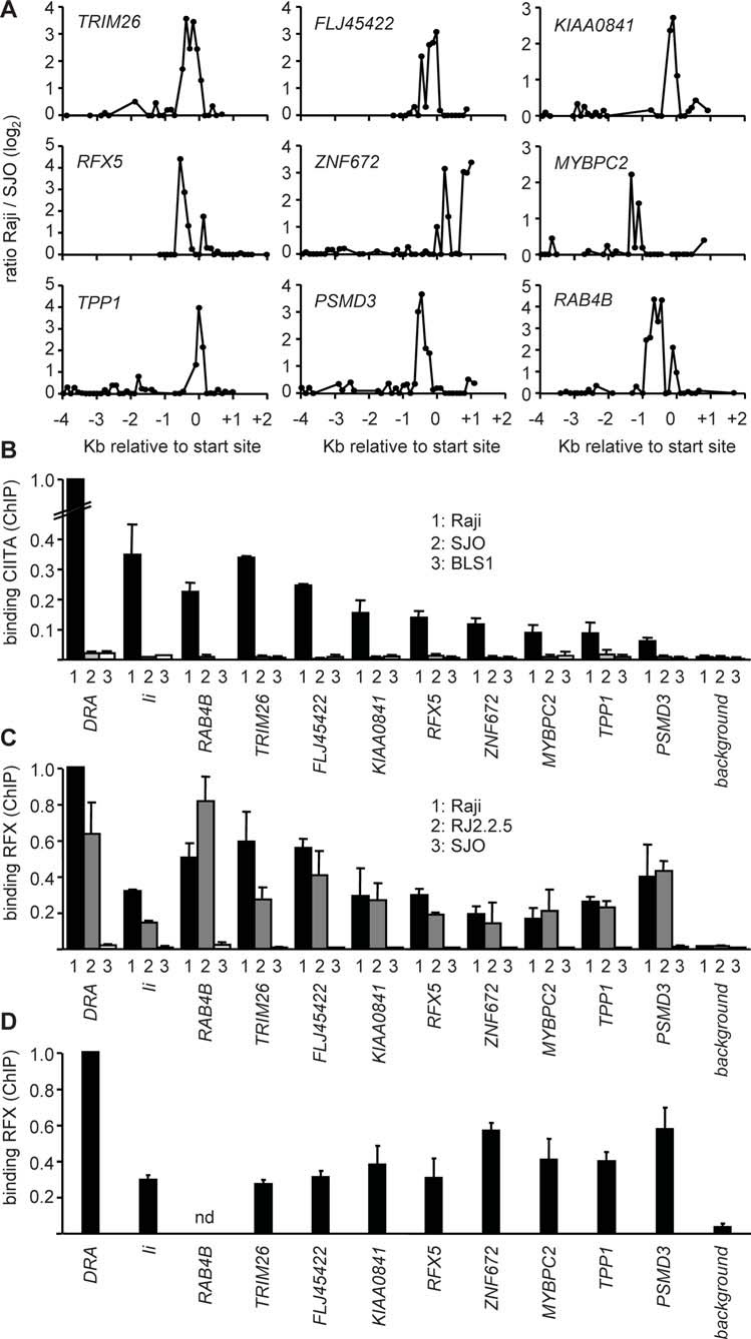
Recruitment of CIITA to the new target genes is dependent on binding of RFX. (A) Representative CIITA binding profiles obtained for the new target genes in ChIP-chip experiments comparing Raji and SJO cells. Results are represented as in Figure 2. (B) Binding of CIITA to the new target genes was assessed by quantitative ChIP experiments performed with Raji, SJO and BLS1 cells. Results are expressed relative to binding of CIITA at *HLA-DRA* in Raji, and show the mean and SD of 3 independent experiments (each performed with triplicate PCR measurements). (C) Binding of RFX to the new target genes was assessed by quantitative ChIP experiments performed with Raji, RJ2.2.5 and SJO cells. Results are expressed relative to binding of RFX at *HLA-DRA* in Raji, and show the mean and SD of 3 independent experiments (each performed with triplicate PCR measurements). (D) Binding of RFX to the new target genes was assessed by quantitative ChIP experiments performed with iDC. Results are expressed relative to binding of RFX at *HLA-DRA*, and show the mean and SD of 3 independent experiments (each performed with triplicate PCR measurements). nd, not done.

At all nine new target genes, strong and reproducible peaks were evident in ChIP-chip experiments comparing CIITA-ChIP probes from Raji and SJO cells (Figure 5A, Table 1). This suggested that recruitment of CIITA to the new genes is – as established for other known target genes – strictly dependent on binding of RFX. To verify this we performed quantitative ChIP experiments comparing binding of CIITA between Raji, RFX5-deficient SJO cells and RFXANK-deficient BLS1 cells. As observed for the control *HLA-DRA* and *Ii* genes, binding of CIITA is completely lost in SJO and BLS1 cells, indicating that it requires an intact RFX complex (Figure 5B).

To document binding of RFX to the new target genes directly, we performed quantitative ChIP experiments with Raji, RJ2.2.5 and SJO cells. As shown previously for well established CIITA regulated genes [30],[31], binding of RFX to the new target genes is strong in Raji, unaffected in RJ2.2.5, but completely abolished in SJO (Figure 5C). Finally, RFX was also found to bind to the new target genes in iDC (Figure 5D) and IFNγ induced cells (data not shown).

The analysis of *RFX5*, *RFXAP* and *RFXANK* mRNA abundance by quantitative RT-PCR demonstrated that the level of RFX expression is very similar in Raji B cells and iDC (data not shown). This is consistent with the observation that the strength of CIITA binding observed in ChIP-chip experiments at validated target genes is very similar between Raji and DC (compare Figures 3B and 4A).

### CIITA Is Recruited to the New Targets via a Typical S-Y Enhancer

**Figure 6:**
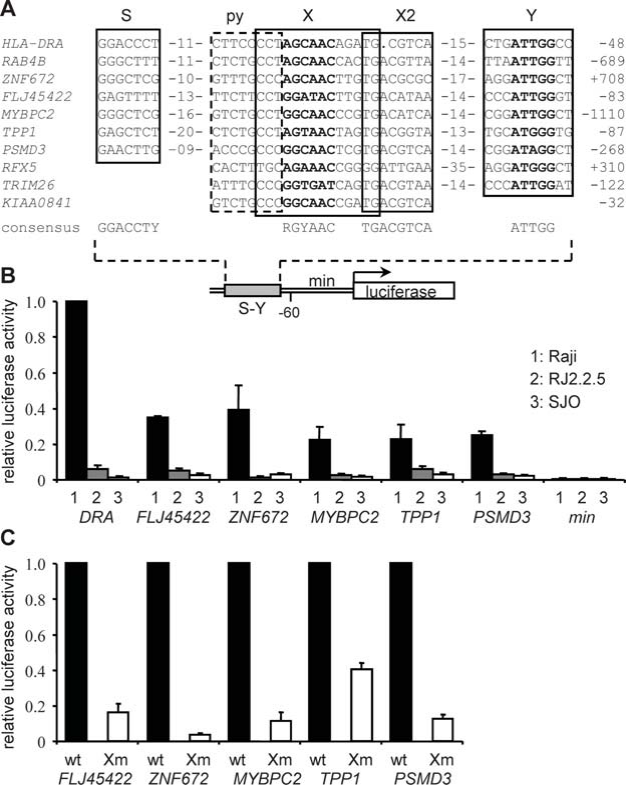
The new target genes contain RFX and CIITA dependent S-Y enhancers. (A) Alignment between the S-Y motif of the *HLA-DRA* gene and sequences found within the regions to which CIITA is recruited at the new target genes. Sequences corresponding to the S, X, X2 and Y elements are boxed. The dashed box indicates a pyrimidine (py) rich region generally found at the 5′ end of the X box. Numbers indicate spacing in nucleotides between the S and py or X2 and Y sequences. S and Y sequences are only shown if homology is evident. (B) Transient transfections were performed with luciferase reporter gene constructs containing the *HLA-DRA* regulatory region or hybrid promoters in which the S-Y motif of *HLA-DRA* was replaced with the S-Y sequences from the indicated new target genes. A construct containing the basal *HLA-DRA* promoter lacking the S-Y module (min) was used as negative control. Constructs were transfected into Raji, RJ2.2.5 and SJO cells. Results are expressed relative to the activity of the *HLA-DRA* construct in Raji, and show the mean and SD of 3 independent experiments (each performed with triplicate luciferase measurements). (C) A mutation disrupting the X box was introduced into the constructs containing the new S-Y enhancers. Activities of the wild type (wt) and mutated (Xm) constructs were compared in Raji cells. Results are expressed relative to the activity of wt construct, and show the mean and SD of 3 independent experiments (each performed with triplicate luciferase measurements).

The finding that recruitment of CIITA to the new target genes is dependent on binding of RFX prompted us to search for sequences resembling the S-Y enhancer. In all *MHC-II* genes, the S, X, X2 and Y boxes of the S-Y enhancer are highly conserved with respect to their order, orientation and spacing (Figure 6A) [7]–[9]. A similar tightly-constrained arrangement of S, X, X2 and Y sequences was evident in six of the new target genes (*RAB4B*, *FLJ45422*, *ZNF672*, *MYBPC2*, *TPP1* and *PSMD3*) at positions lying within the region to which CIITA is recruited (Figure 6A). Homology to a complete S-Y motif was less evident in the remaining three new target genes (*TRIM26*, *KIAA0841* and *RFX5*). Although these three genes do contain a well conserved X/X2 region, no obvious S element is evident and only two of them (*TRIM26* and *RFX5*) have a Y-like sequence (Figure 6A). This prompted us to determine whether NF-Y actually binds to the new S-Y enhancers. Quantitative ChIP experiments performed with an NF-Y antibody confirmed that the new S-Y enhancers, including two of the imperfect ones, are indeed bound by NF-Y (Figure S3). The only gene to which binding of NF-Y was detected only very weakly is the one (*KIAA0841*) lacking a potential Y-like sequence.

To determine whether the new S-Y motifs can function as RFX and CIITA dependent enhancers we generated reporter gene constructs in which the S-Y motif of the *HLA-DRA* promoter was replaced with the S-Y motifs from the new target genes (Figure 6A). The activity of these chimeric constructs, the control *HLA-DRA* construct and a construct driven by a minimal promoter lacking an S-Y module were assessed in Raji, RJ2.2.5 and SJO cells (Figure 6B). In Raji, the constructs containing the new S-Y motifs exhibited activities that were at least 20-fold greater than the basal activity of the minimal promoter and attained levels ranging from 25% to 40% of the activity of the *HLA-DRA* construct. This expression was abolished in RJ2.2.5 and SJO cells. These results confirm that the new S-Y motifs function as transcriptional enhancers regulated by RFX and CIITA. To confirm that the X box is a critical element of the new S-Y enhancers we performed reporter gene assays with constructs having a mutated X box (Figure 6C). The X box mutation strongly decreased activity of the new S-Y enhancers (Figure 6C). We have recently reported similar results for the new S-Y motif of *RAB4B* [32].

### Regulation of the New Target Genes by CIITA

**Figure 7:**
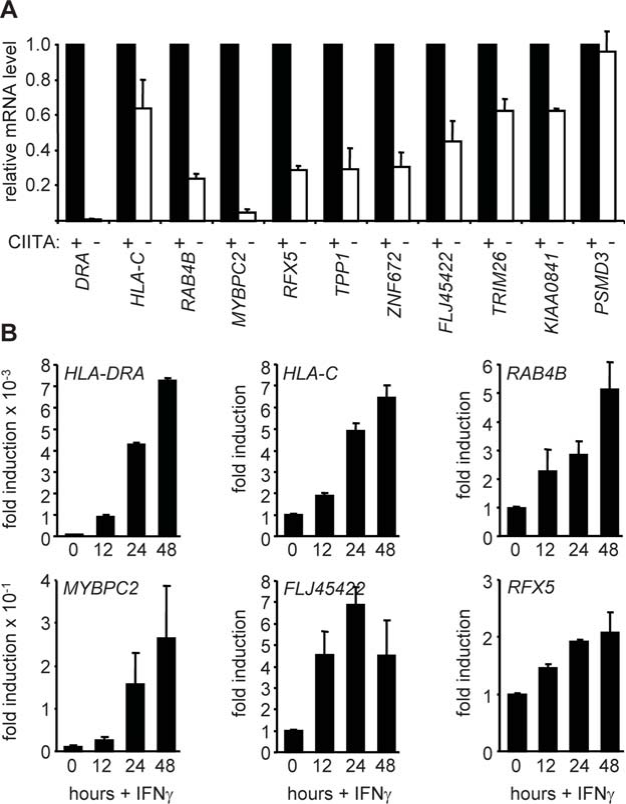
The new target genes are regulated by CIITA in B cells and are induced by IFNγ. (A) mRNA levels for the new target genes were compared between RJ2.2.5 cells (open bars) and RJ2.2.5 cells complemented with a CIITA expression vector (black bars). Results are represented relative to the expression found in the complemented cells, and show the mean and SD of 3 independent experiments (each performed in triplicate PCR measurements). (B) mRNA levels for the indicated target genes were measured in Me67.8 cells induced with IFNγ for 0, 12, 24 and 48 hours. Results are represented as fold induction relative to uninduced cells, and show the mean and SD of 3 independent experiments (each performed in triplicate PCR measurements). *HLA-DRA* and *HLA-C* were included as control CIITA-regulated genes.

To confirm that the novel target genes are regulated by CIITA we quantified their endogenous mRNA abundance by quantitative RT-PCR in RJ2.2.5 cells and in RJ2.2.5 cells complemented with a CIITA expression vector (Figure 7A). Expression of eight of the new target genes (*RAB4B*, *TRIM26*, *FLJ45422*, *KIAA0841*, *RFX5*, *ZNF672*, *MYBPC2* and *TPP1*) was significantly reduced in RJ2.2.5. This reduction was not as strong as that observed for classical targets of CIITA, such as *HLA-DRA*. However, it was similar to or stronger than the reduction observed for *HLA-C*. The new targets thus resemble *MHC-I* genes in that their expression is modulated by, but not strictly dependent on, CIITA [7]–[11].

We next performed real-time RT-PCR experiments with melanoma cells to determine whether expression of the new target genes is induced by IFNγ (Figure 7B). mRNA abundance for four of the new target genes (*RAB4B*, *FLJ45422*, *MYBPC2* and *RFX5*) was increased by IFNγ according to a time course similar to that observed for the control *HLA-DRA* and *HLA-C* genes. Although induction of the new target genes was not as strong as for *HLA-DRA*, it was similar in magnitude to the induction of *HLA-C*. The new targets thus behave like *MHC-I* genes in that they exhibit a significant level of basal expression prior to IFNγ induced CIITA expression [7]–[12].

## Discussion

We have used four ChIP-chip approaches to establish a list of genes regulated directly by CIITA in B cells and DC. In addition to *MHC-II*, *MHC-I* and *Ii* genes, only nine new bona fide target genes were discovered. The current number of genes that have been demonstrated to be regulated directly by binding of CIITA does therefore not exceed twenty five. At all of these genes, the recruitment of CIITA is strictly dependent on binding of RFX to an X box sequence situated near the promoter. In most cases, this X box is situated in a characteristic S-Y enhancer. One of the new target genes encodes the RFX5 subunit of RFX, indicating the existence of a positive feedback loop for enhancing CIITA recruitment. Finally, seven of the nine new target genes are known or likely to be implicated in cellular processes contributing to antigen presentation (see below). Taken together, these results show that CIITA is dedicated for the regulation of a remarkably compact and highly specialized gene expression module devoted to antigen presentation. This is consistent with the fact that all phenotypes documented in BLS patients can be attributed to defects in antigen presentation. Furthermore, the finding that CIITA recruitment is strictly dependent on RFX at all validated target genes is in agreement with the observation CIITA deficient patients can not be distinguished phenotypically from RFX-deficient patients.

Robust and reproducible binding of CIITA was observed at all previously known and newly validated target genes in nine independent ChIP-chip experiments. No other bona fide target genes were identified by any of the four strategies. Genes at which signals were weaker and less reproducible turned out to be false positives. This suggests that most target genes of CIITA have been identified. It is nevertheless possible that certain target genes have been missed. There could be additional targets in specific cell types. In this respect it may be relevant that the *COL1A2* promoter was not found to be occupied by CIITA in B cells or DC (Figure S4 and Figure S5) although earlier ChIP experiments had suggested that CIITA regulates this gene in IFNγ induced cells [22]. It is also possible that certain targets were missed for technical reasons. For example, the relevant CIITA associated sequences could be refractory to PCR amplification, hybridize inefficiently or have been excluded by the NimbleGen array design program. Finally, additional target genes could be controlled by distant CIITA-dependent enhancers that are situated outside of the 5 kb promoter regions present on the NimbleGen arrays. To address the latter possibility we performed ChIP-chip experiments using a high density array of our own design carrying the entire extended human MHC as well as other selected regions of interest, including several of the target genes identified here (see Materials and Methods). ChIP-chip experiments performed with Raji and DC confirmed binding of CIITA to all target genes present on the array but did not - with the exception of known enhancers found in the vicinity of MHC-II genes [36],[37] - lead to the identification of any novel intergenic binding sites (Table S2, Figure S6). This contrasts with the high density of STAT1, p53 and NF-kB binding sites found by ChIP-chip in chromosome 22, many of which are situated far from transcription start sites. Intergenic CIITA-binding sites do thus not appear to be frequent, although we can of course not exclude their existence in other regions of the genome.

The functions of two of the new target genes are unknown. *KIAA0841* encodes a protein containing no characteristic sequence motifs providing clues to its function. *ZNF672* encodes a transcription factor belonging to the Kreuppel zinc-finger family [38]. However, the genes and functions that *ZNF672* might regulate are unknown. For the remaining seven new target genes there is a known or potential link within antigen presentation by MHC-II or MHC-I molecules. *TRIM26* and *FLJ45422* are situated with the class I region of the MHC. For *RAB4B* and *RFX5*, a key role in antigen presentation has already been established. *RFX5* plays a pivotal role in activating MHC-II expression because it encodes the largest DNA-binding subunit of RFX and is essential for recruiting CIITA to its target genes [30]–[33]. *RAB4B* encodes an isoform of the small GTPase RAB4. RAB4 is associated with early and recycling endosomes, and regulates recycling of membranes and proteins from these compartments back to the cell surface [39]. These recycling processes play important roles in various antigen presentation processes, including MHC-II restricted presentation of peptides derived from antigens internalized by receptor-mediated uptake in B cells, cross-presentation of endocytosed antigens by MHC-I molecules in DC and the presentation of intact proteins by DC to the antigen receptors of B cells [40]–[44]. RAB4 has been implicated directly in the MHC-II restricted presentation of antigens internalized by receptor-mediated uptake in B cells [44]. For *MYBPC2*, *TRIM26*, *PSMD3*, *TPP1* and *FLJ45422*, a role in antigen presentation is suggested by the nature of the protein and/or the cellular processes in which they function. *FLJ45422* encodes a protein of unknown function exhibiting similarity to MHC-I molecules. *TPP1* encodes a lysosomal protease [45] that could influence the generation of peptides presented by MHC-II molecules. *PSMD3* encodes a regulatory subunit of the proteasome, a large protease complex implicated in the generation of peptides presented by MHC-I molecules [46]. *MYBPC2* encodes an immunoglobulin superfamily member [47] that can bind to myosin and filamentous actin, and modifies the actin-stimulated ATPase activity of myosin. Although *MYBPC2* is expressed abundantly in muscle and is best known for its role in muscle contraction [48], it is also expressed at lower level in other cell types and its tight regulation by CIITA suggests that it could have additional functions in antigen presenting cells. Importantly, the actin cytoskeleton and/or actin-based myosin motors have been implicated in MHC-II trafficking and receptor-driven antigen presentation in B cells, the formation of immune synapses between antigen presenting cells and T cells, and antigen capture and presentation by MHC-I and MHC-II molecules in DC [49]–[51]. Finally, *TRIM26* encodes a member of the tripartite motif (TRIM) family of ubiquitin E3 ligases [52]. Members of this family are implicated in diverse biological processes. They promote the ubiquitination of specific substrate proteins, thereby controlling their abundance by proteasome mediated degradation or their activity, intracellular trafficking or subcellular localization by proteasome-independent mechanisms. It is tempting to speculate that TRIM26 may regulate either the generation of specific antigenic peptides by the proteasome, or the abundance, activity or subcellular localization of specific proteins implicated in antigen presentation.

The *CIITA* gene is frequently silenced by epigenetic mechanisms in tumors. It has been proposed that the loss of MHC-II expression and/or a reduction in MHC-I expression resulting from the silencing of *CIITA* might allow tumors to evade immune surveillance [23],[53]. Our finding that CIITA is remarkably specific for genes implicated in antigen presentation is consistent with the hypothesis that the association between silencing of *CIITA* and tumorigenicity reflects a reduction in the antigen presentation capacity of the tumor cells. Although there is a well established link between the loss of MHC-I expression and escape from immune surveillance [54], it remains unclear how direct MHC-II mediated antigen presentation by tumor cells contributes to anti-tumor responses *in vivo*. Therefore, an alternative possibility that has to be kept in mind is that defective CIITA expression could represent a selective advantage for tumors because it contributes to the expression of non-MHC genes. In this respect, certain of the new target genes may be relevant. For instance, an altered transcription program due to reduced *ZNF672* expression, deregulated vesicular traffic due to reduced *RAB4B* expression, perturbed intracellular actin-based transport due to reduced *MYBPC2* expression, and altered ubiquitin-dependent degradation or regulation of specific proteins due to lower *TRIM26* expression, could all contribute to the development of tumors.

Two of the new target genes, *RAB4B* and *TPP1*, had been suggested to be regulated by CIITA in earlier studies. Both were among the genes that were found by microarray experiments to be downregulated in CIITA-deficient cells [14]. *RAB4B* was also singled out by a bioinformatic screen designed to identify genes containing S-Y motifs [32]. In contrast, more than 70 other genes suggested previously to be regulated by CIITA [13]–[22] were not found to be direct targets in our ChIP-chip experiments (Figure S4). For several of the most interesting candidates we were moreover unable to confirm binding of CIITA to their promoters by classical ChIP experiments (Figure S5). Finally, in a previous study using CIITA-deficient and CIITA-transgenic mice no direct control of these genes by CIITA could be documented [55]. The influence of CIITA on the expression of these genes is therefore likely to be mediated by indirect mechanisms. For several genes, an indirect mechanism involving sequestration of the general co-activator CBP by CIITA has been proposed [16],[17],[21]. Certain of the genes could be regulated by one of the two transcription factors - RFX5 and ZNF672 - shown here to be controlled by CIITA. Finally, there is growing evidence that ubiquitination and regulatory subunits of the proteasome can play key roles in transcriptional regulation [56]. The modulation of *TRIM26* and *PSMD3* expression by CIITA could thus have indirect impacts on the transcription of certain genes.

The remarkably focused role of CIITA emphasized here contrasts with results derived from large-scale binding studies for other transcription factors. Good illustrations are provided by Foxp3, Stat1, cMyc and p53. Over 1000 Foxp3 target genes were identified by scanning for binding sites in the promoter regions of 16'000 mouse genes [1]. Large scale mapping of Stat1 binding sites in chromosome 22 or selected (ENCODE) regions of the human genome have pointed to hundreds if not thousands of target genes [2],[3]. More than 300 cMyc target sites were identified in chromosomes 21 and 22 [4]. Finally, p53 binds to at least 500 target sites in the human genome [5]. Similar large numbers of targets have been reported for all other transcription factors for which large-scale binding studies have been reported. The high degree of specificity observed here for CIITA is thus unprecedented. Why CIITA presents this unique degree of specificity is unknown. One explanation may reside in the finding that CIITA recruitment appears to be strictly dependent on the assembly of a well-defined multifactor enhanceosome complex on a relatively large (65–70 bp) composite regulatory module (the S-Y motif) that is tightly constrained with respect to its sequence content and architecture [32]. Such S-Y modules are likely to be much less frequent in the genome than binding sites for individual transcription factors. A second explanation may lie in the fact that CIITA has quite an unusual origin for a nuclear transcription factor. It is the only transcriptional activator belonging to the mammalian nucleotide-binding domain and leucine-rich repeat (NLR) containing family, a large group of proteins exerting cytoplasmic functions implicated in cell death, inflammation and innate immunity [57]–[59]. The ability to activate transcription of a specific set of genes in the adaptive immune system may represent a recently evolved specialization acquired by an ancestral NLR protein originally having a completely different cytoplasmic function.

## Materials and Methods

### Cells

Raji, BLS1, SJO and RJ2.2.5 B cells, RJ2.2.5 cells complemented with an expression vector encoding CIITA isoform III [32] and Me67.8 melanoma cells [35] were cultured in RPMI + Glutamax medium complemented with 10% fetal calf serum and antibiotics. Me67.8 cells were induced with 200U/ml IFNγ (Invitrogen). Human monocyte-derived DC were generated and matured with LPS as described [24].

### Chromatin Immunoprecipitation (ChIP)

ChIP experiments were performed as described using antibodies specific for CIITA and RFX [30],[31] or NF-Y (Diagenode). Results were quantified by real-time PCR using the primers listed in Table S3. PCR was performed using the iCycler iQ Real-Time PCR Detection System (Biorad) and a Sybr-Green-based kit for quantitative PCR (iQ Supermix Biorad).

### ChIP-Chip Experiments

CIITA-ChIP samples were verified by quantitative PCR to assess the enrichment of *HLA-DRA* sequences. DNA extracted from the ChIP samples were blunted for 30 minutes at 72°C with 3U of Pfu polymerase (Promega) and phosphorylated with T4 Polynucleotide kinase (New England Biolabs). 120 pmoles of adaptors, consisting of annealed oligonucleotides A (5′-GCGGTGACCCGGGAGATCTGAATTC-3′) and B (5′-GAATTCAGATC-3′), were ligated to the DNA by overnight incubation at 16°C with 2000U of T4 DNA ligase (New England Biolabs). Two rounds of PCR amplification with oligonucleotide A were performed using 1.25U of Taq polymerase (New England Biolabs) and 0.025U of PfuTurbo polymerase (Stratagene). The cycle used was: 1 X (2′ at 55°C, 5′ at 72°C, 2′ at 95°C), 28 X (1′ at 95°C, 1′ at 60°C, 2′ at 72°C), 5′ at 72°C. 4 µg of each DNA were purified and sent to NimbleGen for probe preparation and hybridization to arrays carrying the 5 kb promoter regions (approximately −4 kb to +1 kb relative to the transcription start site) of 27434 human genes, or to a custom array of our own design. The latter carries all unique sequences from the entire extended human MHC (7.7 Mb on chromosome 6, genomic coordinates 26.1 Mb to 33.8 Mb on hg17) as well as a number of other selected regions (total of 0.9 Mb), including several of the target genes identified here. These genomic regions are covered at high density with overlapping Tm-matched oligonucleotides (∼50 bp long) spaced such that their 5′ ends are situated ∼10 bp apart.

### Analysis of ChIP-Chip Data

Data sets from five independent experiments (three Raji/RJ2.2.5 and two iDC/mDC comparisons) were analyzed with SignalMap software (NimbleGen). Positive peaks in the test/control signal ratios were calculated using a 500 base-pair sliding window and a cut-off that was deliberately set at a very low value (5% of maximum) to minimize the risk of eliminating weak peaks. This generated a large number of potential peaks (11262, 13322 and 15980 in the three Raji/RJ2.2.5 experiments, 13017 and 11795 in the two iDC/mDC experiments. The following procedure was then used to identify peaks present reproducibly in the five experiments. First, all peaks found in the five experiments were merged into a single list and sorted according to their genomic midpoint coordinates. A sliding window of five consecutive peaks was then used to identify genes exhibiting a peak in each of the five experiments. For these genes, the distance was calculated between the midpoints of the first and fifth peak and the list of these genes was then resorted according to these calculated peak proximities. Since the likelihood that peaks detected in different experiments correspond to the same binding site will increase with increased peak proximity, candidates with the smallest distance values between peaks were considered to be the most likely candidates. Similar algorithms were used to identify peaks present in only four or three of the five experiments. Finally, to avoid the risk of eliminating target genes because of a threshold difference between B cells and DC, all genes exhibiting closely superposable peaks in only the three Raji/RJ2.2.5 experiments or only the two iDC/mDC experiments were retained as candidates. Peaks for approximately 500 candidate genes identified by this procedure were re-examined visually and assigned a score based on reproducibility, width and strength of the signals. A score of 3 was assigned to genes where strong signals (log_2_ ratios exceeding 2) were spread over at least 400 base-pairs and were present in at least four experiments. A score of 2 was assigned to genes where strong signals were present in at least three experiments, or when weaker or narrower peaks were present in four or five experiments. A score of 1 was assigned to genes where three experiments exhibited weak signals or when signals were detected in only two experiments. A score of 0 was assigned to genes that did not meet the above criteria. To simplify representation of the results in the Figures, negative test/control signal ratios were set at log_2_  =  0.

Two alternative approaches were also used to analyze the ChIP-chip data. One was a published method [34]. The second consisted of the following unsupervised approach. Peaks from the three Raji/RJ2.2.5 and two iDC/mDC experiments were pooled together, sorted by chromosome, midpoint coordinate and experiment, and considered to correspond to the same binding site if their distance was < 750 bp. Peaks that were only positive in 1 or 2 experiments were excluded from the following steps. To identify groups in the remaining peaks, they were represented as 4 bit binary vectors with the first 2 bits representing the number of peaks with significant log_2_ ratios and the last 2 bits representing the number of positive experiments. Log_2_ ratios were considered significant if their values were above 0.66, which corresponds to the quantile for a probability of 0.95 calculated on the complete set of log_2_ ratios determined over all experiments and peaks. Since the binary variables are asymmetric, a distance matrix between any pair of peaks represented by the rows of the binary matrix is calculated using the Jaccard distance [60]. This distance measure ranges from 0 (closely related peaks) to 1 (unrelated peaks). The resulting distance matrix was then used to determine the number of groups (partitions) using the PAM (Partitioning Around Medoids) algorithm [61]. Based on the above distance matrix, this algorithm calculates all possible partitions ranging from 2 to n-1 subgroups. For each partition the overall average silhouette width [62] is calculated and the partition that maximizes it is considered optimal.

### Plasmids and Reporter Gene Assays

S-Y motifs were amplified by PCR and cloned in the *HLA-DRA* luciferase plasmid described previously [31]. The X box was altered to AAGCTACCACTCGT by site directed mutagenesis as described [32]. This mutation has a major impact on the activity of known S-Y enhancers. Transfections were done by electroporation. Dual luciferase reporter gene assays were performed according to instructions from the manufacturer (Promega).

### mRNA Quantification

RNA extraction and cDNA synthesis were done as described [31]. Quantification was done by real-time PCR using the primers listed in Table S4. Results were normalized using 18S rRNA. Results were confirmed with several primer pairs.

## Supporting Information

Figure S1 CIITA binding profiles are shown for three well known target genes, *HLA-DRA* (left column), *HLA-DRB1* (middle column) and *HLA-DMA* (right column). The ChIP-chip profiles are derived from three Raji/RJ2.2.5, one Raji/input, three Raji/SJO and two iDC/mDC experiments. Results are represented as log_2_ ratios between the hybridization signals obtained with the test probes (CIITA-ChIP samples from Raji or iDC) and the control probes (input DNA or CIITA-ChIP samples from RJ2.2.5, SJO or mDC). Each dot corresponds to a single oligonucleotide on the array. The dotted lines in the *HLA-DRA* profile of the Raji/input experiment and the *HLA-DMA* profile of the third Raji/SJO experiment indicate sporadic peaks probably representing LM-PCR amplification artifacts. The schematic maps above the profiles show positions of the transcription start sites (arrows) and S-Y enhancers (grey boxes). The scale in Kb relative to the transcription start site is provided below.
(0.52 MB PDF)

Figure S2 ChIP-chip results are shown for six control genes (*GAPDH*, *ACTB*, *PTPRC*, *CD19*, *ITGAX*, and *LY75*) that are not regulated by CIITA. Representative profiles from Raji/RJ2.2.5 (left column), iDC/mDC (middle column) and Raji/SJO (right column) experiments are shown. Results are represented as log_2_ ratios between the hybridization signals obtained with the test probes (CIITA-ChIP samples from Raji or iDC) and the control probes (CIITA-ChIP samples from RJ2.2.5, mDC or SJO). Each dot corresponds to a single oligonucleotide on the array. The dotted lines in the iDC/mDC profiles shown for *ACTB* and *LY75* indicate sporadic peaks probably representing LM-PCR amplification artifacts. The scale in Kb relative to the transcription start site is provided below.
(0.40 MB PDF)

Figure S3 Binding of NF-Y to the indicated new target genes was assessed by quantitative ChIP experiments performed with Raji cells. Results are expressed relative to binding of NF-Y at *HLA-DRA*. As negative control we used a sequence exhibiting only nonspecific CIITA association (background). Results show the mean and SD of 2 independent experiments (each performed with triplicate PCR measurements).
(0.01 MB PDF)

Figure S4 ChIP-chip results are shown for eight genes (*COL1A2*, *EIF3I*, *FASLG*, *IL4*, *KPNA6*, *RBBP4*, *YARS*, and *PLXNA1*) previously suggested to be regulated by CIITA. Representative profiles from Raji/RJ2.2.5 (left column), iDC/mDC (middle column) and Raji/SJO (right column) experiments are shown. Results are represented as log_2_ ratios between the hybridization signals obtained with the test probes (CIITA-ChIP samples from Raji or iDC) and the control probes (CIITA-ChIP samples from RJ2.2.5, mDC or SJO). Each dot corresponds to a single oligonucleotide on the array. The scale in Kb relative to the transcription start site is provided below.
(0.42 MB PDF)

Figure S5 Binding of CIITA to the *IL4*, *FASLG*, *KPNA6*, *YARS*, *EIF31*, *PLXNA1*, *RBBP4*, and *COL1A2*, genes was assessed by quantitative ChIP experiments performed with Raji (R) and RJ2.2.5 (RJ) cells. Results are expressed relative to binding of CIITA at *HLA-DRA* in Raji. As negative control we used a sequence exhibiting only nonspecific CIITA association (background). Two different *COL1A2* primer pairs were tested. Results show the mean of triplicate PCR measurements obtained for a representative experiment.
(0.01 MB PDF)

Figure S6 CIITA-ChIP-chip experiments performed with a high density custom array. (A) Representative CIITA binding profiles are shown for *HLA-DRA*, *HLA-DMA*, *FLJ45422*, and *TRIM26* (top four profiles) and for candidate distant sequences A and B (bottom two profiles). Results are represented as log_2_ ratios between the hybridization signals obtained with CIITA-ChIP samples from Raji and RJ2.2.5. The scale in Kb relative to the transcription start site (arrows) is provided for the four genes. For the potential distant binding sites the nucleotide coordinates on chromosome 6 are indicated. (B) Binding of CIITA to the *HLA-DRA* gene and to the two potential binding sites A and B were assessed by quantitative ChIP experiments performed with Raji (R) and RJ2.2.5 (RJ) cells. Results are expressed relative to binding of CIITA at *HLA-DRA* in Raji. As negative control we used a sequence exhibiting only nonspecific CIITA association (background).
(0.05 MB PDF)

Table S1 Score 1 genes identified by CIITA-ChIP-chip experiments.
(0.09 MB PDF)

Table S2 ChIP-chip experiments performed with a custom high-density MHC array.
(0.10 MB PDF)

Table S3 Primer sequences used for ChIP.
(0.01 MB PDF)

Table S4 Primer sequences used for real-time RT-PCR.
(0.08 MB PDF)
