# Supplementary figures and images for: Identification of CIITA Regulated Genetic Module Dedicated for Antigen Presentation
Source: PLoS Genet. 2008 Apr 25;4(4):e1000058. doi: 10.1371/journal.pgen.1000058 (PMC2278383; doi:10.1371/journal.pgen.1000058)

**Figure S1**

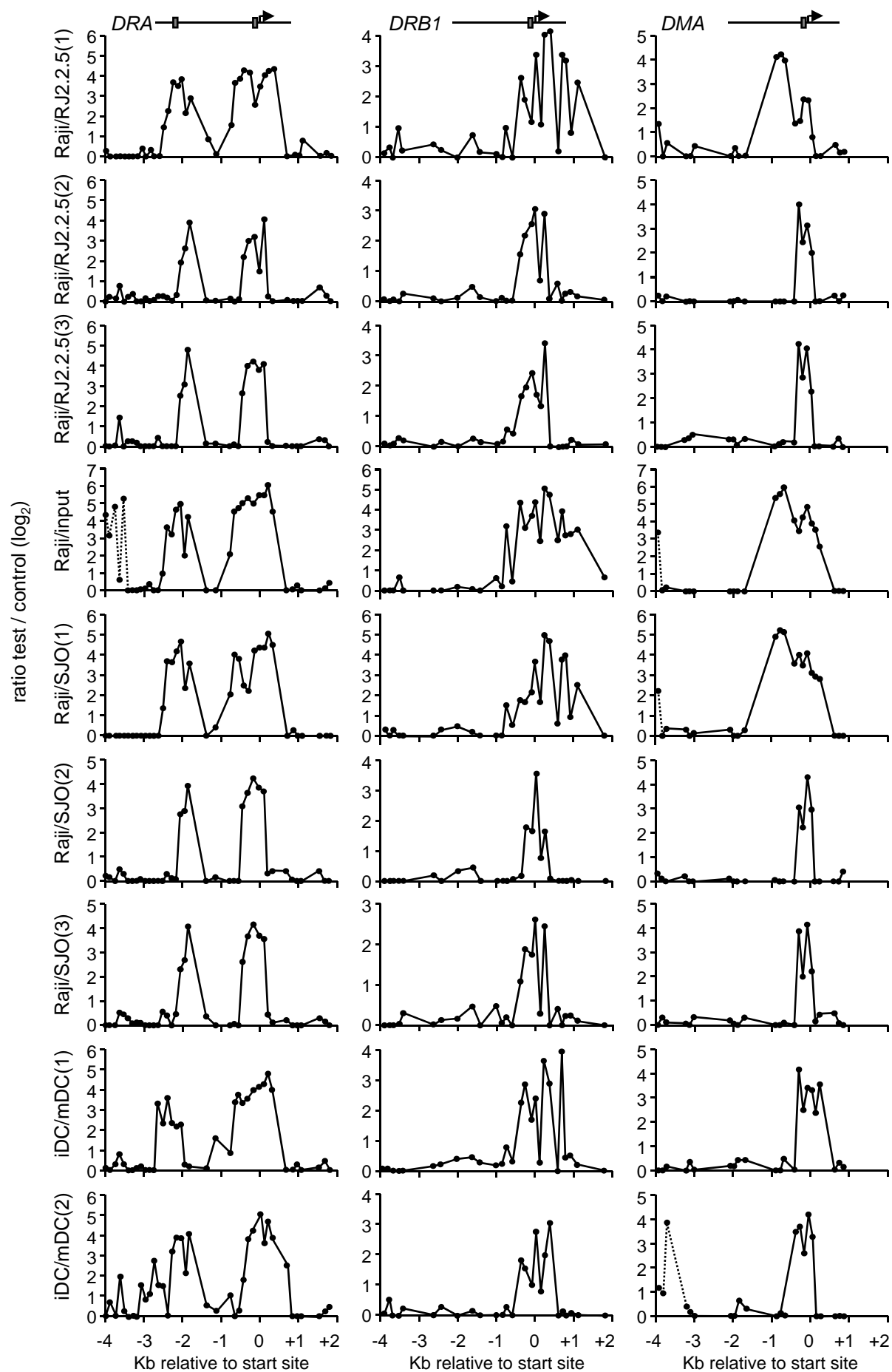

Supplement: Figure S1 — CIITA binding profiles are shown for three well known target genes, HLA-DRA (left column), HLA-DRB1 (middle column) and HLA-DMA (right column). The ChIP-chip profiles are derived from three Raji/RJ2.2.5, one Raji/input, three Raji/SJO and two iDC/mDC experiments. Results are represented as log2 ratios between the hybridization signals obtained with the test probes (CIITA-ChIP samples from Raji or iDC) and the control probes (input DNA or CIITA-ChIP samples from RJ2.2.5, SJO or mDC). Each dot corresponds to a single oligonucleotide on the array. The dotted lines in the HLA-DRA profile of the Raji/input experiment and the HLA-DMA profile of the third Raji/SJO experiment indicate sporadic peaks probably representing LM-PCR amplification artifacts. The schematic maps above the profiles show positions of the transcription start sites (arrows) and S-Y enhancers (grey boxes). The scale in Kb relative to the transcription start site is provided below. (0.52 MB PDF) [file pgen.1000058.s001.pdf]

Figure S2

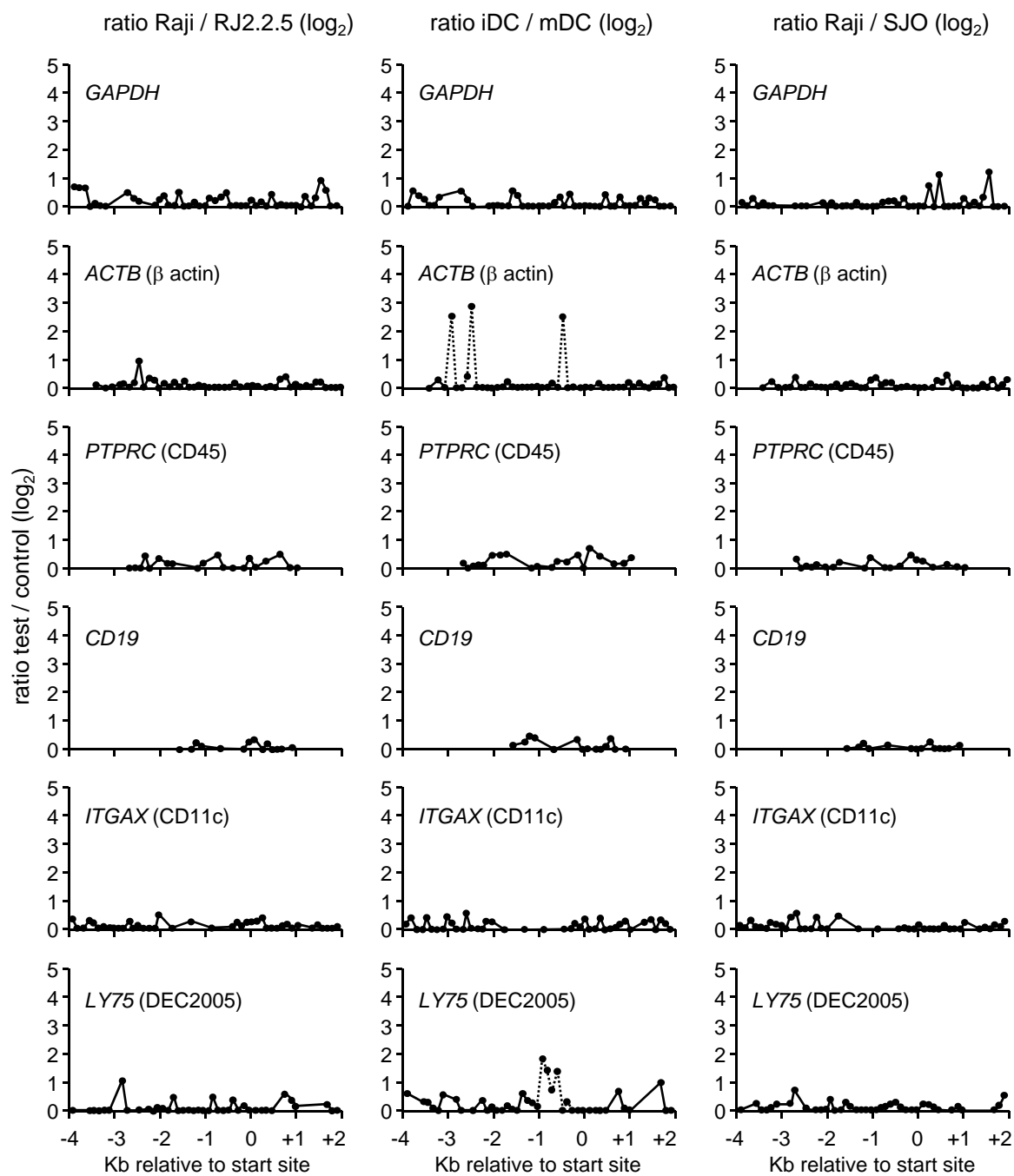

Supplement: Figure S2 — ChIP-chip results are shown for six control genes (GAPDH, ACTB, PTPRC, CD19, ITGAX, and LY75) that are not regulated by CIITA. Representative profiles from Raji/RJ2.2.5 (left column), iDC/mDC (middle column) and Raji/SJO (right column) experiments are shown. Results are represented as log2 ratios between the hybridization signals obtained with the test probes (CIITA-ChIP samples from Raji or iDC) and the control probes (CIITA-ChIP samples from RJ2.2.5, mDC or SJO). Each dot corresponds to a single oligonucleotide on the array. The dotted lines in the iDC/mDC profiles shown for ACTB and LY75 indicate sporadic peaks probably representing LM-PCR amplification artifacts. The scale in Kb relative to the transcription start site is provided below. (0.40 MB PDF) [file pgen.1000058.s002.pdf]

**Figure S3**

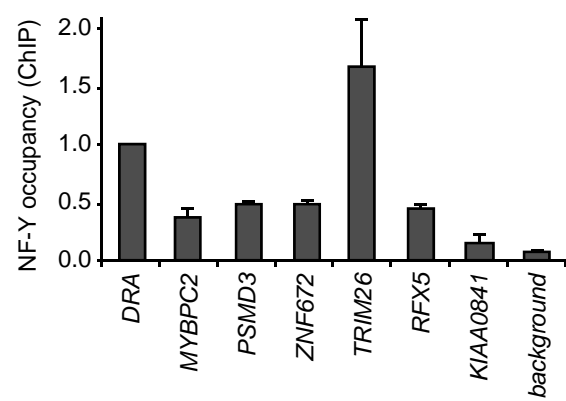

Supplement: Figure S3 — Binding of NF-Y to the indicated new target genes was assessed by quantitative ChIP experiments performed with Raji cells. Results are expressed relative to binding of NF-Y at HLA-DRA. As negative control we used a sequence exhibiting only nonspecific CIITA association (background). Results show the mean and SD of 2 independent experiments (each performed with triplicate PCR measurements). (0.01 MB PDF) [file pgen.1000058.s003.pdf]

**Figure S4**

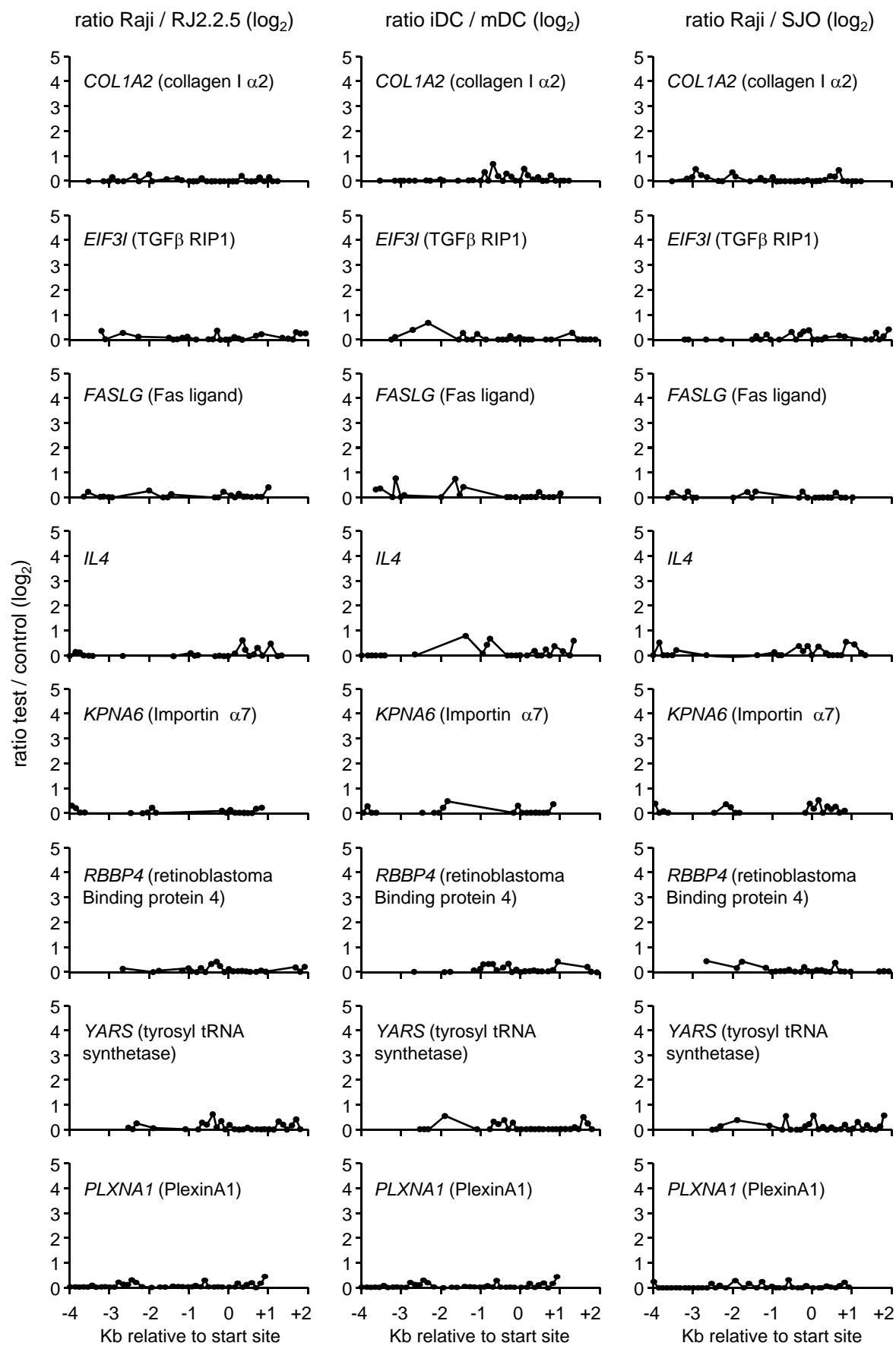

Supplement: Figure S4 — ChIP-chip results are shown for eight genes (COL1A2, EIF3I, FASLG, IL4, KPNA6, RBBP4, YARS, and PLXNA1) previously suggested to be regulated by CIITA. Representative profiles from Raji/RJ2.2.5 (left column), iDC/mDC (middle column) and Raji/SJO (right column) experiments are shown. Results are represented as log2 ratios between the hybridization signals obtained with the test probes (CIITA-ChIP samples from Raji or iDC) and the control probes (CIITA-ChIP samples from RJ2.2.5, mDC or SJO). Each dot corresponds to a single oligonucleotide on the array. The scale in Kb relative to the transcription start site is provided below. (0.42 MB PDF) [file pgen.1000058.s004.pdf]

Figure S5

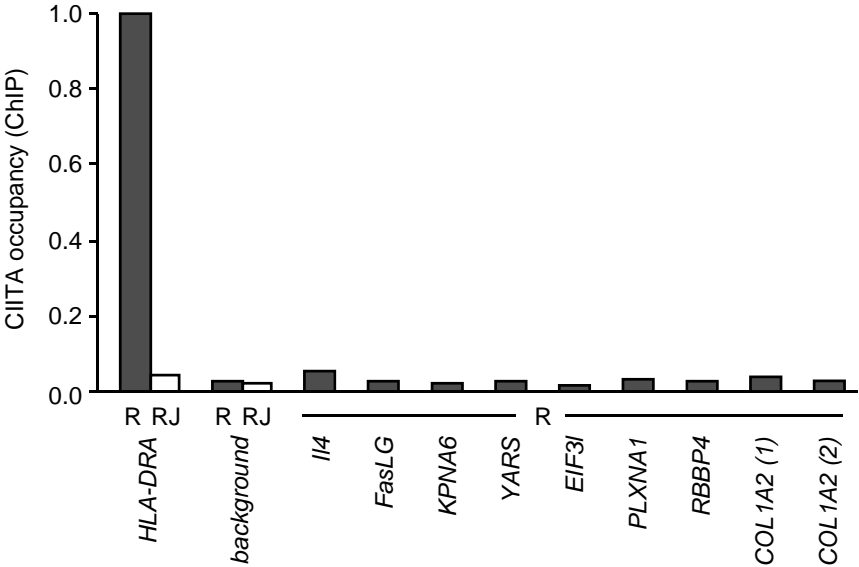

Supplement: Figure S5 — Binding of CIITA to the IL4, FASLG, KPNA6, YARS, EIF31, PLXNA1, RBBP4, and COL1A2, genes was assessed by quantitative ChIP experiments performed with Raji (R) and RJ2.2.5 (RJ) cells. Results are expressed relative to binding of CIITA at HLA-DRA in Raji. As negative control we used a sequence exhibiting only nonspecific CIITA association (background). Two different COL1A2 primer pairs were tested. Results show the mean of triplicate PCR measurements obtained for a representative experiment. (0.01 MB PDF) [file pgen.1000058.s005.pdf]

Figure S6

**A.**

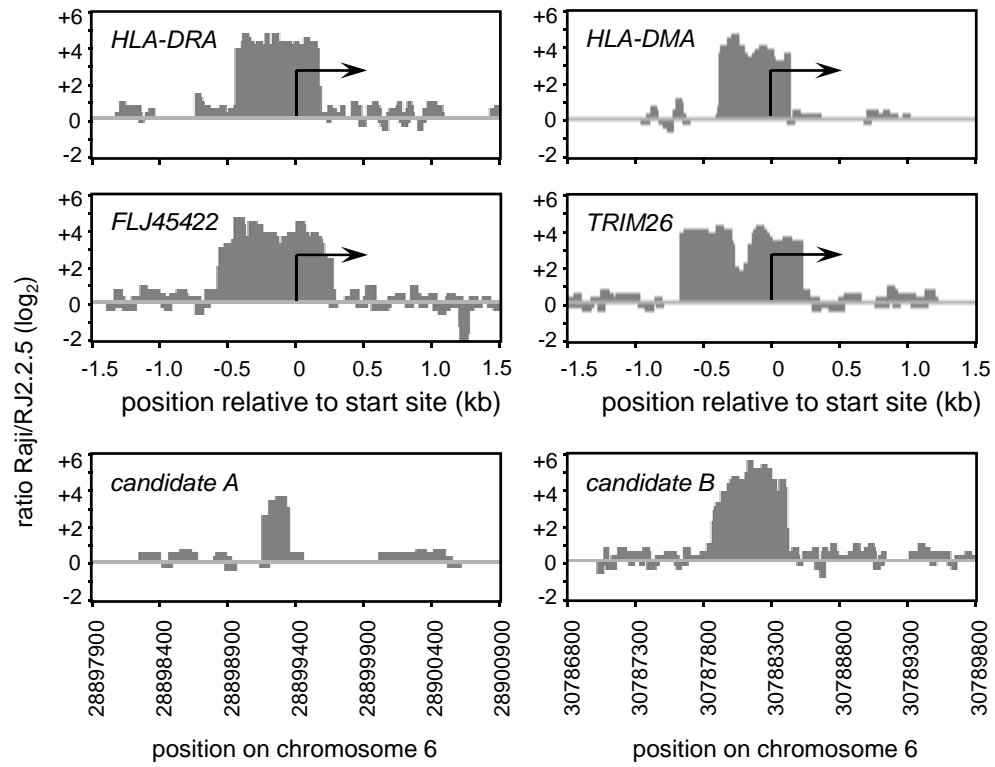

**B.**

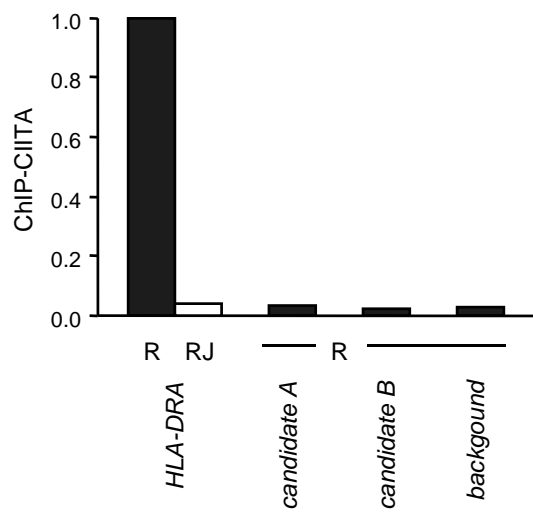

Supplement: Figure S6 — CIITA-ChIP-chip experiments performed with a high density custom array. (A) Representative CIITA binding profiles are shown for HLA-DRA, HLA-DMA, FLJ45422, and TRIM26 (top four profiles) and for candidate distant sequences A and B (bottom two profiles). Results are represented as log2 ratios between the hybridization signals obtained with CIITA-ChIP samples from Raji and RJ2.2.5. The scale in Kb relative to the transcription start site (arrows) is provided for the four genes. For the potential distant binding sites the nucleotide coordinates on chromosome 6 are indicated. (B) Binding of CIITA to the HLA-DRA gene and to the two potential binding sites A and B were assessed by quantitative ChIP experiments performed with Raji (R) and RJ2.2.5 (RJ) cells. Results are expressed relative to binding of CIITA at HLA-DRA in Raji. As negative control we used a sequence exhibiting only nonspecific CIITA association (background). (0.05 MB PDF) [file pgen.1000058.s006.pdf]
