# Supplementary material for: Identification of CIITA Regulated Genetic Module Dedicated for Antigen Presentation
Source: PLoS Genet. 2008 Apr 25;4(4):e1000058. doi: 10.1371/journal.pgen.1000058 (PMC2278383; doi:10.1371/journal.pgen.1000058)
Supplement: Table S1 — Score 1 genes identified by CIITA-ChIP-chip experiments. (0.09 MB PDF) [file pgen.1000058.s007.pdf]

Table S1. Score 1 genes identified by CIITA-ChIP-chip experiments.

gene	accession	validation by ChIP[1]		gene	accession	validation by ChIP[1]
AK092544	AK092544	nt		*LRRC51*	NM_145309	nt
AK124820	AK124820	nt		*MARCO*	NM_006770	nt
AP1S2	NM_003916	negative		*MAT2A*	NM_005911	nt
AY010114	AY010114	nt		*MGC52000*	BC073913	nt
BTN2A2	NM_006995	nt		*MLL5*	NM_182931	negative
C4A	NM_007293	negative		*MRPL47*	NM_020409	nt
CACYBP	NM_014412	nt		*MSH3*	NM_002439	nt
CALML3	NM_005185	negative		*MYLK*	NM_053025	nt
CEP63	NM_025180	negative		*NBPF11*	NM_183372	nt
CKMT1	NM_001015001	nt		*NBPF14*	NM_015383	nt
CNOT7	NM_054026	nt		*NIN*	NM_182944	nt
COG5	NM_006348	nt		*NPIP*	NM_006985	negative
CR749856	CR749856	nt		*NPL*	NM_030769	nt
DLX6	NM_005222	nt		*OR5AY1*	NM_001004732	nt
DVL2	NM_004422	negative		*PARVG*	NM_022141	nt
ECSIT	NM_016581	nt		*PHACS*	NM_032592	nt
ENO1	NM_001428	nt		*PLOD3*	NM_001084	nt
FOS	NM_005252	nt		*PSMA2*	NM_002787	nt
GCUD2	NM_207418	nt		*RBM12B*	NM_203390	nt
GORASP1	NM_031899	nt		*RNASE7*	NM_032572	nt
GTPBP3	NM_133644	nt		*SFRS2*	NM_003016	negative
IGHA1	AK128476	nt		*SGK*	NM_005627	nt
IMAA	AB040413	negative		*TLR4*	NM_138554	nt
IQCH	NM_001031715	nt		*TMED1*	NM_006858	nt
JMJD1A	NM_018433	negative		*TMEM14C*	NM_016462	nt
KIR3DL1	NM_013289	nt		*TNFRSF9*	NM_001561	nt
KIR2DL3	NM_014511	nt		*TRAF2*	NM_021138	nt
KIR2DS4	NM_012314	nt		*TTC11*	NM_016068	nt
KRT14	NM_000526	nt		*ZFAND3*	NM_021943	nt
KRTAP5-9	NM_005553	nt				

[1]Results of classical ChIP experiments; negative, no detectable binding of CIITA; nt, not tested.
