# Supplementary material for: Identification of CIITA Regulated Genetic Module Dedicated for Antigen Presentation
Source: PLoS Genet. 2008 Apr 25;4(4):e1000058. doi: 10.1371/journal.pgen.1000058 (PMC2278383; doi:10.1371/journal.pgen.1000058)
Supplement: Table S2 — ChIP-chip experiments performed with a custom high-density MHC array. (0.10 MB PDF) [file pgen.1000058.s008.pdf]

**Table S2.** ChIP-chip experiments performed with a custom high-density MHC array.

| | gene | accession number | ChIP-chip Raji/RJ2.2.5[1] | | ChIP-chip iDC/mDC[1] | | validation by ChIP[2] |
|---|---|---|---|---|---|---|---|
| | | | 1 | 2 | 1 | 2 | |
| **MHCII genes** | *DRA* | NM_019111 | +++ | +++ | +++ | +++ | **positive** |
| | *DRB1* | NM_002124 | +++ | +++ | +++ | +++ | nt |
| | *DRB5* | NM_002125 | +++ | +++ | +++ | +++ | nt |
| | *DQB* | NM_002123 | ++ | ++ | ++ | +++ | nt |
| | *DOA* | NM_002119 | ++ | + | +++ | + | **positive** |
| | *DOB* | NM_002120 | +++ | +++ | + | +++ | **positive** |
| | *DMA* | NM_006120 | +++ | +++ | +++ | +++ | nt |
| | *DMB* | NM_002118 | +++ | +++ | +++ | +++ | **positive** |
| | *DPA* | NM_033554 | + | ++ | +++ | +++ | **positive** |
| | *DPB* | NM_002121 | + | ++ | +++ | +++ | **positive** |
| | *Ii* | NM_004355 | +++ | +++ | +++ | +++ | **positive** |
| **Newly identified genes** | *RAB4B* | NM_016154 | +++ | +++ | +++ | +++ | **positive** |
| | *TRIM26* | NM_019111 | +++ | +++ | ++ | + | **positive** |
| | *FLJ45422* | NM_001004349 | +++ | +++ | + | | **positive** |
| | *TPP1* | NM_000391 | ++ | ++ | ++ | +++ | **positive** |
| | candidate | nucleotide position[3] | | | | | |
| **Putative distal binding sites** | *A* | 28896391 | + | + | + | | negative |
| | *B* | 30787523 | +++ | +++ | ++ | | negative |

[1]Results of independent CIITA-ChIP-chip experiments performed with a high density custom array: + signs indicate the presence of a peak in the signal ratios; number of + signs refer to quality of peaks.

[2]Validation of CIITA binding by classical ChIP experiments: positive, confirmed binding; negative, absence of binding; nt, not tested.

[3]Nucleotide coordinates on chromosome 6, human genome build 17
