# Supplementary material for: Identification of CIITA Regulated Genetic Module Dedicated for Antigen Presentation
Source: PLoS Genet. 2008 Apr 25;4(4):e1000058. doi: 10.1371/journal.pgen.1000058 (PMC2278383; doi:10.1371/journal.pgen.1000058)
Supplement: Table S3 — Primer sequences used for ChIP. (0.01 MB PDF) [file pgen.1000058.s009.pdf]

Table S3. Primer sequences used for ChIP[1].

Genes	Forward	Reverse
Background	CTGCCCTAGCACATTTGTTAACG	GCTGGTCTGCTGGAATAGATCC
HLA-DRA	ATTTTTCTGATTGGCCAAAGAGTAATT	AAAAGAAAAGAGAATGTGGGGTGTAA
Ii	GTATTTCCAGCCTTTGTAGCTTTCAC	TGGAGAGGAATCTGATTCGTCC
RAB4B	AAACCTAAGCAGCCCAAC	TTATATCCTCCGCGTAGTCC
TRIM26	GGTCCCGCCTGATTTCAGTTTC	TCACTGATCACCGGGCAAATCC
FLJ45422	GTGACATAATCCCACTTCTCAC	AGTGCCGGGATCGTAGTGCC
KIA0841	AGTGAGCGGGAGAACGAG	CGGTCAGGCTGGGTCTTG
RFX5	GGTGGCTACGAGGTAGTTTC	GCTCTCCTCCCAACTCATC
ZNF672	TCCCAGTCTTCCCAGCAACC	CGTCGGCTAGTGGTCAGTCT
MYBPC2	GCACAGAGGGTTCCGTTTATG	AGGGCTCAATTCAGAGGGAAG
TPP1	CAGTGGGAGCTACGTTGTC	CGGGAGAAAGGGATCACGTTG
SCYLBP1	GTTGCTCCTTCCCAAATACC	CATTCGTTTCCTCTGGCTTACC
BMF	GAGCCAGTCCTGCAACAAC	AGGTCCCAGCTCCACTGAAC
LRPPRC	GCTGCCATTGCTCGAACG	TACCCTGCGGTCACATGC
RALA	GCTGCATCTCCTTTCTGTTCG	CAGCGTGAAGAATGGATAGC
PSMD3	GTTGAGGCGCTCTTAAAGTC	TCCTCTCGAGTGCATTTCTG
BRD2	TGGCCAATGGAGGAGCTAC	AAGAGGTGCGGAAGCCATCG
TRIM14	CACGACTCCACTGGATTG	AGCCTTTCTCCGGTACTG
TMS9F	CAAGGACGGACAGAACTACG	GGCAAGGGACAGTTGAACTC
C1or151	CAGTGCAGTGTTGAAGCTTG	CTGGGCATCAGAGTGATACC
MACF1	CTCTGGGAGGACAAACAAGATT	AATGAGGCACAGAGTTTAGGTA
LEMD2	AGGGCAGGTGATTTGGTTAC	GGCCGGCAATAAATGTTTGG
DENND1A	CAGCACACCCACCCTTTC	GGGCATTGGGACCCTTTG
CHML	GCAGAGCACCAATCTCTTCAAC	CAGAAGAAGCCATCACTCTTCG
ZFYVE19	GGAGTGGAGAGGCTTTGAG	CGCTTCTGCCTTGACTGTG
ANKRA2	TTTGCCGCCTTTACGCTC	TGACCCAGAACCGTCAAC
AB007983	CTGCTGGGCTTCTGCTTTG	AATCAAGGCGCGAAAGGACC
RUFY2	TAGCAGGTACGGTCTTAGGATG	ATTGGACGGCTTCTTCTGTG
TDH	ATTCCCTGACCTGGCAACATC	TGGCACCTGGCACTTAATGAG
FLJ44082	TATGATCCCTGTGCCTCTAC	TCCTCTTCTGCTTCCTTCTG
LOC125893	ACCTTGCCACATTCATTACAC	GTGATTCATGCCTTGCAAAC
NPIP	CTGAGAGGTGCACAGTGTC	GCTCCTTCCTCACGTTCTCAG
IMAA	TCCGCTGTTCAGATCATGG	CGGCATTTAACCCTGGAACC
CALM3	GGACCTGCTGATTGCTTTG	GGACTAACGGCTCTGAATTG
MLL5	TCCCACGTCAGGGAACTAC	GCTGTTGGTGGAGAAGGTTTG
JMJD1A	CTGCACCAGGGGCCTTTTC	CAACCGCAGAGGGAGTGAG
DVL2	CCAGTGTGGCCCAAAGTAG	GATTTCGGGTGAGCGCAAG
SRF2	TTAGGAGGCAAGTATGGATGAC	ATTATGTATCGCATGGGTAGGG
Cep63	TCGTCTTGCCTTGAGGATTTG	TTAGGCACAGGAGCTCATGAAC
AP1S2	CCCTGTCGCCGTGCTGAGGAAG	GCCTAAGCCTGCCCTATGC
C4A	TACCGGGAGCTGCTCCTATC	GAGGCCAGGGAAAGACTCAC
EIF3S2	AACGCCGCTGTCTTCTAC	AGGTTTCGAGGTCACTTC
FASL	CCTGTAGCTGGGAGCAGTTC	GTTGCTGTCGCTGTGCTACC
IL4	GGAGAGTCTGCCTGTTATTC	AAGTACAGGTGGCATCTTGG
KPNA6	GGTGTAATGATAGGCGATTGGG	GACAATATGGCGGATCTGTAGG
RBBP4	GAGTGTGCGGACCAAACCTCTC	AGCCGACTTCCAGCGATTGC
YARS	GTTGCTGGCGGGAAGATAG	ACCACCGTCCTCAGATCTC
PlexinA1	CAGCTTATAGGGCCTCAG	AGTGTGAGCCCTTCCAGTG
Col1A2 (1)	CGGCTAAGTTGGAGGTACTG	GTCCGCGTATCCACAAAG
Col1A2 (2)	CTCCACCCTACAAGTGGCCTAC	GCAGATCTCCGGCGATCAAAGC

[1]Sequences are provided in the 5’-3’ orientation.
