# Supplementary material for: Identification of CIITA Regulated Genetic Module Dedicated for Antigen Presentation
Source: PLoS Genet. 2008 Apr 25;4(4):e1000058. doi: 10.1371/journal.pgen.1000058 (PMC2278383; doi:10.1371/journal.pgen.1000058)
Supplement: Table S4 — Primer sequences used for real-time RT-PCR. (0.08 MB PDF) [file pgen.1000058.s010.pdf]

Table S4. Primer sequences used for real-time RT-PCR[1].

mRNA	Forward	Reverse
HLA-DRA	GCCAACCTGGAAATCATGACA	AGGGCTGTTCGTGAGCACA
HLA-C	CAGAAGTACAAGCGCCAGG	TAGGCGGACTGGTCATACC
RAB4B	GGTGGGAAGACTGTGAAG	GTAATAACTCCGCGTCACTG
TRIM26	TTCATTCCAGCCTCGCAGTC	GCTCCAGCACTCAGTCAATC
FLJ45422	CAGTACTCTGATGCATCTCAT	TGCTGCTGTCAGAGATGAG
KIA0841	CCTCTGGTGCTGGGATCTAC	CTCAGGGATTCGCTCCAGTG
RFX5	GCCAAGTACACTGAGCAATG	GCAACAGGCAAGACTCTCAC
ZNF672	CGGTGTCTGAGGAGGCAGTC	AGTCCCAAGGCACGCTCTTC
MYBPC2	AGGTCGGTGATGCCATACTG	AGTGGCCTGTTGAAGAAGAG
TPP1	TCTCTGGCAAATGCAGTTAC	AAGGTGAGACTCAGCTCTTC
PSMD3	ATCACGCCCGGGTCTATGAG	ATGCCGAAGCGTAGCTGTCC
18S	CTTAGAGGGACAAGTGGCG	ACGCTGAGCCAGTCAGTGTA
RFXANK	GAACCGGATGCCAGTGTTTC	AGAGTGGTGGAGTGCTTCAG
RFXAP	GTCAAACTCGAGGAAAGTGCA	TTCTGGACTTCTTAGTAACGACAGTCT

[1]Sequences are provided in the 5'-3' orientation.
